# Biogenic Selenium Nanoparticles in Biomedical Sciences: Properties, Current Trends, Novel Opportunities and Emerging Challenges in Theranostic Nanomedicine

**DOI:** 10.3390/nano13030424

**Published:** 2023-01-19

**Authors:** Marjorie C. Zambonino, Ernesto Mateo Quizhpe, Lynda Mouheb, Ashiqur Rahman, Spiros N. Agathos, Si Amar Dahoumane

**Affiliations:** 1 School of Biological Sciences and Engineering, Yachay Tech University, Hacienda San José s/n, San Miguel de Urcuquí 100119, Ecuador; 2 Laboratoire de Recherche de Chimie Appliquée et de Génie Chimique, Hasnaoua I, Université Mouloud Mammeri, BP 17 RP, Tizi-Ouzou 15000, Algeria; 3 Center for Midstream Management and Science, Lamar University, 211 Redbird Ln., Beaumont, TX 77710, USA; 4 Earth and Life Institute, Catholic University of Louvain, B-1348 Louvain-la-Neuve, Belgium; 5 Department of Chemical Engineering, Polytechnique Montréal, C.P. 6079, Succ. Centre-Ville, Montréal, QC H3C 3A7, Canada; 6 Department of Chemistry and Biochemistry, Université de Moncton, 18, Ave Antonine-Maillet, Moncton, NB E1A 3E9, Canada

**Keywords:** nanomedicine, SeNPs, biosynthesis, selenoproteins, pharmacokinetics, theranostics, biomedical applications

## Abstract

Selenium is an important dietary supplement and an essential trace element incorporated into selenoproteins with growth-modulating properties and cytotoxic mechanisms of action. However, different compounds of selenium usually possess a narrow nutritional or therapeutic window with a low degree of absorption and delicate safety margins, depending on the dose and the chemical form in which they are provided to the organism. Hence, selenium nanoparticles (SeNPs) are emerging as a novel therapeutic and diagnostic platform with decreased toxicity and the capacity to enhance the biological properties of Se-based compounds. Consistent with the exciting possibilities offered by nanotechnology in the diagnosis, treatment, and prevention of diseases, SeNPs are useful tools in current biomedical research with exceptional benefits as potential therapeutics, with enhanced bioavailability, improved targeting, and effectiveness against oxidative stress and inflammation-mediated disorders. In view of the need for developing eco-friendly, inexpensive, simple, and high-throughput biomedical agents that can also ally with theranostic purposes and exhibit negligible side effects, biogenic SeNPs are receiving special attention. The present manuscript aims to be a reference in its kind by providing the readership with a thorough and comprehensive review that emphasizes the current, yet expanding, possibilities offered by biogenic SeNPs in the biomedical field and the promise they hold among selenium-derived products to, eventually, elicit future developments. First, the present review recalls the physiological importance of selenium as an oligo-element and introduces the unique biological, physicochemical, optoelectronic, and catalytic properties of Se nanomaterials. Then, it addresses the significance of nanosizing on pharmacological activity (pharmacokinetics and pharmacodynamics) and cellular interactions of SeNPs. Importantly, it discusses in detail the role of biosynthesized SeNPs as innovative theranostic agents for personalized nanomedicine-based therapies. Finally, this review explores the role of biogenic SeNPs in the ongoing context of the SARS-CoV-2 pandemic and presents key prospects in translational nanomedicine.

## 1. Introduction

Nanotechnology deals with the design of particles, tools and devices in the range between 1 and 100 nm in size with specific functions at the cellular, atomic and molecular levels [1,2]. Nanomedicine is a relatively new but fast-developing field that can potentially elicit a major impact on human health by combining nanotechnology-based techniques and methods with biomedical and pharmaceutical sciences [3,4,5,6,7]. Thus, nanotechnology can remarkably assist the therapy, diagnosis, monitoring and control of biological systems for future development of personalized medicine with tailored and optimized treatments [8,9,10]. Nanomedicine embraces nanopharmaceuticals, nanoimaging, sensing, plus diagnostics and therapy, also known as theranostics [11,12,13,14,15,16,17]. The interdisciplinary fields of nanotechnology and nanomedicine have been propelled to the forefront in investigations from academia, the pharmaceutical industry, clinical organizations, and several national and international funding and regulatory agencies [18,19].

**Figure 1:**
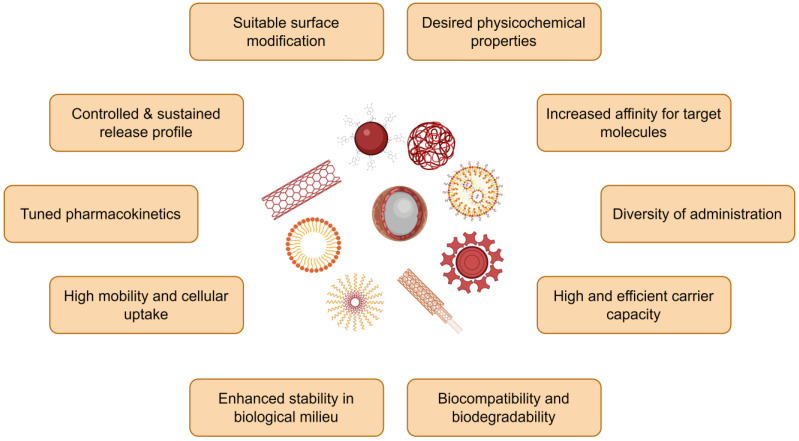
Main advantages of nanoparticles in biomedicine. Explored nanoplatforms in the biomedical field might be organic, inorganic, or hybrid. Inorganic NPs might be of different compositions, such as core-shell quantum dots and passivated inorganic NPs by an organic/polymeric layer, and of various shapes, such as spheres and nanotubes. Liposomes, polymeric NPs, and biopolymer-based NPs are some examples of organic NPs.

Nanoparticles (NPs) exhibit unique properties over bulk materials, such as small size, high surface area, low polydispersity, colloidal stability, tunable surface charge and chemistry, easy modification, and multi-functionality (Figure 1) [20,21,22,23]. In recent years, NPs have been developed to harness their biological interactions at the molecular and cellular levels with a high degree of specificity towards diagnosing and treating several diseases [24,25,26,27,28,29,30]. The utilization of NPs has opened new therapeutic opportunities for agents which otherwise cannot be used effectively as traditional drug formulations due to their poor bioavailability and instability [31]. In particular, inorganic NPs, with their superior intrinsic chemical, biological, and magnetic properties, have been designed for several diagnostic [32,33,34], therapeutic [35,36], health [37], and agricultural [38,39] applications. Several nanomaterials hold great promise for integrating diagnostic and therapeutic applications, such as monitoring the biodistribution and accumulation at target site, observing and quantifying drug release, and longitudinally evaluating therapeutic efficacy [32,40].

Selenium nanoparticles (SeNPs) have attracted special interest since selenium is an essential micronutrient for the proper functioning of human and animal organisms in trace amounts [41]. Selenium is an important dietary constituent of at least 25 human selenoproteins and enzymes containing selenocysteine [41,42,43]. In the environment, selenium, whose chemical symbol is Se, exists under various oxidation states (2−, 0, 2+, 4+, 6+) and forms, such as selenate (Na_2_SeO_4_), selenite (Na_2_SeO_3_), selenomethionine (SeMet), selenocysteine (SeCys), and solid-state, zerovalent Se (Se^0^) [44,45]. Nanoscale selenium has attracted a great deal of interest worldwide due to its high degree of absorption, great bioactivity, low toxicity, and high efficiency in preventing oxidative damage compared to its organic and inorganic counterparts [46,47]. SeNPs possess remarkable anticancer [48,49,50], antioxidant [51], antidiabetic [52,53], antibacterial, and antibiofilm properties [54,55,56]. Equally noteworthy are the diagnostic applications of SeNPs in nanosensors, cellular imaging, epigenetics, and immunochromatography [34,57,58]. SeNPs can be fabricated through different physical, chemical, and biological techniques [59]. Nevertheless, the biological synthesis route, which relies on bacteria, yeast, plants, and microalgae, constitutes a widely explored green alternative that has witnessed tremendous developments and is especially suitable for SeNPs [42,60,61,62,63]. Bionanotechnology, a subset of green nanotechnology, is described as a set of methods that eliminate or reduce the utilization of toxic substances, resulting in cost-effective and eco-friendly alternatives [64,65]. In this sense, biogenic SeNPs are highly biocompatible and stable, due to natural coatings provided by biomolecules that, besides avoiding the use of chemical stabilizers, prevent the aggregation of the particles, improve their pharmacological activity, and protect them against physical and chemical degradation [66,67,68,69].

The translation of Se-based nanotechnology into clinical applications requires not only the development of safe, simple, sustainable, and cost-effective methods for the synthesis of SeNPs, but also a thorough understanding of their relevant physicochemical and biological properties that, in turn, might impact their in vitro and in vivo effects, safety control mechanisms, pharmacokinetics and pharmacodynamics, and potential biomedical applications. Research progress in biogenic SeNPs has implemented a safe-by-design principle, ensuring safety for both human health and the environment. However, this body of research that encompasses research articles and reviews has only discussed individual aspects of biogenic SeNPs, such as the synthesis, properties, and specific biomedical applications. Therefore, this study aims at filling this gap by providing a comprehensive and up-to-date review of the theranostics applications of biosynthesized SeNPs and their potential in translational nanomedicine. Furthermore, the properties, synthesis, and pharmacological activity of SeNPs focused on the molecular mechanisms, cellular interactions, and roles of selenoproteins are presented. Additionally, examples of biocidal and biomedical applications of biogenic SeNPs are detailed. Finally, key aspects that should be addressed to pave the way for clinical applications of biogenic SeNPs are discussed.

## 2. Selenium and Nanoselenium: General Information

Nanotechnology is emerging as an extraordinary platform of technical solutions for complex medical challenges. Nanomedicine involves nanotherapeutics [2], nanopharmaceuticals [70], nanoimaging [71], and theranostics [36]. Compared to conventional medicine, precision nanomedicine offers great physical and biological benefits, such as enhanced efficacy, improved pharmacokinetics and safety, reduced toxicity, and increased tissue selectivity of drug formulations [4,72]. Particularly, the major issue, in the case of Se, in the translation from bench to bedside, is the narrow window from therapeutic effect to toxicity due to the small margin of dosage error [73]. As mentioned above, selenium is an essential micronutrient, playing an important role in endocrine, reproductive, cardiovascular, and immune processes. It also acts as a pleiotropic agent associated with biotherapy and drug delivery for a better immune response and cancer prevention [74].

In addition to its above-mentioned inorganic and organic forms, Se is the principal constituent of some selenoenzymes, such as glutathione peroxidases (GPXs), thioredoxin reductases (TrxR), and deiodinases (DIO), which are essential in biochemical reactions of biological defense systems, including antioxidant activity [75]. Se-biogenic compounds can be found in living organisms in the form of methylated species, selenoamino acids, selenoproteins, selenium peptides, selenoenzymes, selenoamino carboxylic acids, and selenium derivatives of pyrimidines, purines, coenzyme A, cholines, and steroids, among others [76]. Most of these molecules play an important role in the organism’s defense against oxidative stress [77]. They also exhibit remarkable antioxidant and pro-oxidant effects limited by the dose, life span, chemical form of Se compound, route of administration, and oxidation state [78]. Table 1 classifies selenium compounds.

**Figure 2:**
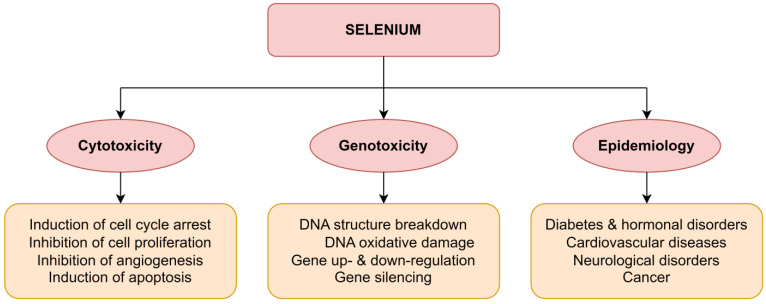
Selenium toxicity in humans and animals: cytotoxicity, genotoxicity, and epidemiology.

Usually, conventional selenium dietary supplements exhibit a low degree of absorption and enhanced toxicity [58]. Therefore, selenium is viewed as a controversial nutrient since high doses are toxic, provoking death, whereas selenium deficiencies can yield chronic and fatal health issues, such as diabetes, thyroid dysfunction, arthrophyma, Keshan disease, and cognitive problems [74,80]. Selenium toxicity has been tracked for decades, showing that low levels elicit efficacious anticarcinogenic activity while high levels can generate carcinogenesis, cytotoxicity, and genotoxicity (Figure 2) [81,82]. Indeed, there is some scientific consensus that the high pro-oxidant property of different redox-active forms of selenium compounds is the key factor to efficiently and selectively combat cancer [82,83,84,85]. For example, methyl selenocysteine (MeSeCys) and methyl selenic acid (MeSeA) were found to be potential anticarcinogenic selenocompounds with little toxicity and high bioavailability as indicated by the increased glycoprotein selenoprotein P (SEPP) biosynthesis [86]. Cao et al. reported the remarkable antitumor activity of MeSeCys in preclinical trials when combined with four different cytostatic drugs (cyclophosphamide, cisplatin, oxaliplatin, and irinotecan), offering protection against organ-specific toxicity [87]. Moreover, selenocysteine, a naturally occurring selenoamino acid, may be a promising anticancer candidate, as it enhances the apoptosis of the A375 human melanoma cell line when combined with 5-fluorouracil [88].

To date, the anticancer activity of selenium is not yet fully demonstrated, especially its in vivo therapeutic efficacy. The main challenge lies in delivering specific concentrations of redox-active selenium directly to the target site (tumor or metastatic cells) to bring about the cytotoxic effect [89]. In the quest of innovative systems to upgrade the bioavailability and the controlled release of selenium, much attention has been focused on SeNPs, which have appeared as the answer to the toxicological concerns due to their novel properties, including high specific surface area, high degree of absorption, and low toxicity when compared to their inorganic and organic analogs [46,58]. As the alternative to elemental zero-valent selenium (Se^0^), nanoscale selenium offers the advantage of a significantly low toxicity without affecting its ability to upregulate selenoenzymes at nutritional levels and induce phase II enzymes at supra-nutritional levels [90]. Nanoscale selenium is bright red, highly stable, and soluble; it has been processed for pharmaceutical and medical applications due to its anticancer, antimicrobial, antioxidant, and antidiabetic activity [62,91]. When released from the NP surface, the unstable Se^0^ atoms are readily oxidized to inactive forms. To prevent this instability, proteins and polysaccharides are mainly used as nanocarriers; these include chitosan [51,92], egg white lysozyme [93], β-lactoglobulin [94], acacia gum, and carboxymethyl cellulose [95].

SeNPs have been integrated in simultaneous treatments involving immunotherapy, chemotherapy, and radiotherapy because they not only possess a sensitive response to radiation stimuli but also exhibit excellent anticancer activity and immune checkpoint inhibitor effect [96]. Indeed, SeNPs deliver the chemotherapeutic drug doxorubicin (DOX) to tumor sites by systemic administration, thereby exerting immunomodulatory activity by enhancing natural killer (NK) cell function [96]. Moreover, SeNPs can effectively increment the persistence of cytokine-induced killer (CIK) cells in peripheral blood in the body. For instance, the combination of SeNPs and CIK cells induces natural killer cells to infiltrate into tumors, and shapes tumor-associated macrophages to trigger powerful immune responses for effective cancer immunotherapy [35]. In addition, SeNPs enable signal transduction from the lysosomes to the nucleus and further potentiate γδ T cell anti-tumor cytotoxicity, promote the production of surface receptors present at the immune cells, typically NKG2D, CD16, CD44, and IFN-γ, and inhibit the expression of PD-1 receptors [97].

**Figure 3:**
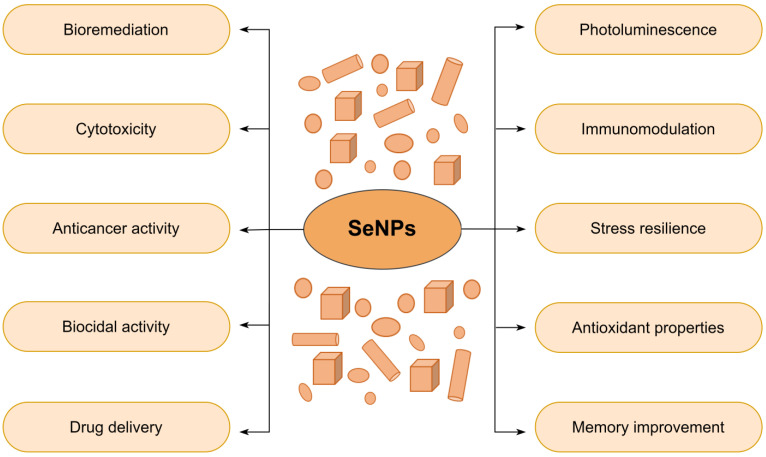
Main biomedical properties and applications of SeNPs.

The effects of SeNPs at the cellular and tissue levels have been thoroughly investigated in, for example, type 2 diabetes mellitus (T2DM) treatment [73], immune and antioxidative responses [98,99], atherosclerosis treatment [100], and semen quality and testis ultrastructure studies [101]. In addition, several studies indicate that nanostructured selenium is gaining attention among dietary supplements and therapeutic agents [76]. In addition, its immunostimulatory [63] and protective effects against heavy metal intoxication [102,103,104] are well-documented. For instance, Sheiha et al. reported the effects of nanoscale selenium supplementation on the growth performance, kidney and liver functions, carcass traits, antioxidant indices, and inflammatory cytokines of growing rabbits subjected to thermal stress [105]. Moreover, Tran et al. combined the carcinostatic activity of Se nanoclusters with the mechanical properties of titanium to build a new anticancer bone implant [106]. Furthermore, Bartůněk et al. evaluated the use of PEGylated SeNPs for specific antimicrobial coatings [107]. Lastly, SeNPs were used as coatings to inhibit biofilm formation [56,108,109]. Figure 3 summarizes key properties and applications of SeNPs [58].

## 3. Selenium Nanoparticles: Methods of Synthesis

Owing to their unique surface activity and particle dispersion, SeNPs offer several advantages over bulk selenium-based materials, such as larger biological activity, higher catalytic efficacy, greater bioavailability, and lower toxicity [42]. The ultimate outlook of precision nanomedicine has consisted in designing and constructing smart NPs for clinical translation. However, the ability to fabricate NPs free of any toxic or hazardous substances is very challenging, especially for applications in nanomedicine. This quest has driven the evolution of different approaches to synthesize SeNPs.

### 3.1. Physical Methods

Physical techniques include, to name a few, microwave irradiation, ultraviolet irradiation, laser ablation, and ultrasonic field treatment [91]. Bright red selenium nanoballs, nanotubes and multi-armed nanorods, with diameters ranging from 20 to 130 nm, were obtained via a microwave approach in which the L-asparagine/H_2_SeO_3_ concentration ratio and the irradiation time controlled the NP diameter and morphology [110]. Using a similar method, pure hexagonal phase SeNPs were obtained with selenium tetrachloride as the precursor under microwave irradiation [111]. Relying on γ-rays, water-stable SeNPs are produced in the presence of various natural macromolecules, such as citrus pectin, sodium alginate, chitosan, and aqueous extract of fermented fenugreek (*Trigonella foenum-graecum*) powder [112]. Moreover, SeNPs were synthesized via nanosecond pulsed laser ablation by irradiating selenium pellets while chitosan acted as the capping agent [113]. Other studies reported the fabrication of selenium nanostructures via laser ablation and discussed their antibacterial activity [114,115,116]. Moreover, cubic-like SeNPs were fabricated by employing a self-assembly process [117]. In addition, SeNPs were obtained via sonochemistry [118]. Physical procedures offer advantages over chemical ones since the latter may require a final calcination step that makes them unsuitable for targeted applications [58].

### 3.2. Chemical Methods

The chemical synthesis of SeNPs is the most conventional and widespread method, which comprises the reduction of metal salts using chemical reducing agents in aqueous or organic media [59]. In addition, the inclusion of stabilizers (e.g., polysaccharides) in the reaction mixture enables the size and shape control of the NPs, ensures their colloidal stability, and avoids their aggregation; hence, the stabilizing molecules are of paramount importance [119]. Basically, chemical methods constitute a time-saving strategy but can be highly expensive and environmentally harmful. Several studies have detailed the chemical synthesis of SeNPs [48,54,120]. For instance, a solution-phase approach yielded monodisperse spherical SeNPs of 20 nm by reducing selenous acid solution using ascorbic acid and water-soluble polysaccharides, such as chitosan, konjac glucomannan, acacia gum, and carboxymethyl cellulose [95]. Size-controlled, stable, and positively charged SeNPs, with sizes ranging from 30 to 150 nm, were fabricated using selenous acid and chitosan to build special chain-shaped intermediates to enhance the cellular uptake and anticancer activity [121]. Another study yielded uniform spherical SeNPs of 103 nm through the reduction of selenic acid with ascorbic acid in the presence of chitosan [92]. These nanostructures continuously grew via a “bottom–up” approach and “top–down” shrinkage processes, generating a stable nanosystem towards pH and enzyme treatment. On the other hand, Chung et al. reported on the synthesis of bovine serum albumin (BSA)-coated selenium nanoparticles (BSA-SeNPs) of 20–800 nm through a one-step reaction relying on sodium selenite, ascorbic acid, and BSA [122]. During the fabrication process, the agitation speed (100 rpm, 400 rpm, and 600 rpm) and sodium selenite to ascorbic acid molar ratios (1:2, 1:4, 1:5, 1:6) impacted the NP size. Another study described the concomitant reduction of sodium selenite by glutathione, and the formation of SeNPs with an effective antibacterial activity of over 99% when coating polyvinyl chloride endotracheal tube substrates [123]. Monodisperse, spherical SeNPs of 46 nm in size were obtained by reducing SeCl_4_ in the presence of ascorbic acid [124]. A facile and versatile electrochemical approach produced spherical SeNPs of 43–85 nm in size using selenium powder doped-carbon paste electrode [125]. A simple wet chemical method relying on sodium selenosulfate in ionic liquid resulted in spherical SeNPs of 76–150 nm in diameter [126].

The use of chitosan (CTS) as an effective material to promote NP production has been extensively investigated owing to its exceptional properties in terms of biocompatibility, biodegradation, and enzymatic resistance [127]. This enables its various applications in the biomedical field, such as in tissue engineering, drug delivery, wound healing, and gene therapy [128]. For example, spherical SeNPs were embedded into CTS microspheres through a spray-drying method for selenium oral delivery with a high efficiency and good biosafety [129]. Selenium nanoencapsulation within CTS networks exhibited decreased toxicity, enhanced antioxidant activity, and controlled in vitro release [130]. The effects of selenium-loaded CTS nanoparticles were assessed in terms of cellular selenium retention, cell survival, and DNA damage in response to selenium exposure, giving rise to novel selenium delivery systems with high specificity and low toxicity for dietary and therapeutic applications [127]. To highlight the promise of SeNPs in cancer treatment, 105-nm SeNPs modified with ferulic acid were synthesized via a simple, low-cost approach to investigate their antitumor activity and DNA-binding affinity [131]. Lastly, a novel, potentially scalable room-temperature procedure allowed the fabrication of SeNPs using selenium oxide as the precursor and lignosulfonate as the stabilizer [132].

### 3.3. Biological Methods

The synthesis of SeNPs via biological methods is receiving increased attention. This green and sustainable synthesis overcomes several drawbacks, including the cost, complexity, and toxicity concerns, and improves the effectiveness of the process [60]. In this regard, several organisms, such as plants, algae, fungi, and bacteria, have been examined in the biogenesis of SeNPs. For example, Medina et al. fabricated spherical SeNPs of 90–150 nm using *Staphylococcus aureus*, methicillin-resistant *S. aureus* (MRSA), *Escherichia coli* and *Pseudomonas aeruginosa*, and found that the antibacterial potential of SeNPs made using a specific bacterium was more efficient against the same bacterial species [133]. Lampis et al. used *Stenotrophomonas maltophilia* to synthesize SeNPs of 160–250 nm, depending on the age of the cultures [134]. Kora et al. isolated the selenite-reducing bacterium *Bacillus cereus* from a lake contaminated by industrial waste to fabricate amorphous and spherical SeNPs of 93 nm [135]. Khoei et al. produced biogenic SeNPs via intra- and extra-cellular pathways using two strains of *Burkholderia fungorum*; this formation was attributed to cytoplasmic enzymatic activation mediated by electron donors [136]. In addition, Kamnev et al. detailed the extracellular production of SeNPs through selenite reduction by living biomass of the rhizobacterium *Azospirillum brasilense* [137].

In the same vein, fungi are also efficient Se-reducing organisms, able to synthesize Se^0^ as well as Se-methylated compounds [60,138,139]. Various fungi have been explored for the green fabrication of SeNPs [140,141,142,143,144,145]. For instance, *Pleurotus ostreatus*-treated aqueous extract of fermented powdered fenugreek seeds was an effective capping and reducing agent to produce SeNPs due to the high amounts of amino acids, proteins, and other reducing agents [112]. Another study reported that the cell wall, cytoplasm, and proteins of *Mariannaea* sp. provided templates for the reduction of Se(IV) to Se(0) through various detoxification mechanisms [146]. Mycogenic SeNPs were obtained from three fractions of *Trichoderma atroviride*: namely, culture filtrate, cell lysate, and cell wall debris; these NPs exhibited antifungal activity against several phytopathogens [147]. A simple and efficient method relying on *Cordyceps sinensis* exopolysaccharides yielded well-dispersed and stable SeNPs [148]. Monodispersed SeNPs of ~22 nm in size, synthesized by *P. chrysogenum* filtrate, were incorporated within carbon nanotubes under γ-irradiation [108]. Similarly, the aqueous extract of *Aspergillus oryzae*-fermented lupin was found to reduce Se ions into spherical, isotropic, and poly-dispersed SeNPs under γ-irradiation [149].

On the other hand, some researchers favor yeast-mediated biosynthesis of metal NPs since yeast biomass production is simple, easy to obtain and scalable [60]. For example, several strains of *Saccharomyces* reduced selenium anions to subsequently form SeNPs [150,151,152]. A suitable green analytical procedure using the yeast *S. boulardii* enabled the direct monitoring of SeNPs synthesis yield [151]. In the same vein, the genetically engineered metal-resistant strain of *Pichia pastoris* is an efficient nanofactory for intracellular SeNP biosynthesis [153]. Finally, the yeast *Nematospora coryli* gives rise to intracellular spherical SeNPs of 50–250 nm that display potential anti-*Candida* and antioxidant activities [154].

Plants have been extensively used for SeNP biosynthesis [155,156,157,158,159]. For instance, *Withania somnifera* possesses constituents, such as alkaloids, flavonoids, phenolics, tannins, and terpenoids, which act as good bio-reductants and capping agents for the synthesis of SeNPs [160]. Phenolic and alcoholic compounds present in guava (*Psidium guajava*) leaf extract are responsible for the synthesis of SeNPs and their stabilization [161]. *Cassia auriculata* leaf extract reduces selenite ions into SeNPs [162]. Lemon [163], prickly pear [164], *Abelmoschus esculents* [165], orange peel [166] *Macleaya cordata* [167], and *Hibiscus sabdariffa* [168], are examples of plants involved in the biosynthesis of SeNPs. In addition, a facile single-step and green in situ method relying on the novel RTFP-3 polysaccharide, extracted from *Rosa roxburghii* fruit, enabled the production of size-controlled and stable SeNPs [169]. In addition to some of the previously mentioned biological properties, biogenic SeNPs synthesized using *Clausena dentata* leaf extract exhibit a strong, dose-dependent mosquito larvicidal activity [170].

## 4. Properties of Selenium Nanoparticles

The morphology, size, and properties (i.e., physical, chemical, biological) of nanomaterials are determined by different factors and reaction parameters, such as synthesis techniques, starting materials, specific surfactants or additives, pH, reaction time, reaction temperature, and the nature of the solvent [171,172]. This section reviews several physicochemical, optoelectronic, catalytic, and biological properties of SeNPs that will help understand their biomedical applications.

### 4.1. Physicochemical Properties

Selenium (Se) is a metalloid that possesses intermediate properties between a metal and a non-metal. It is stable and is not oxidized at room temperature [43]. Selenium shares several chemical and physical properties with its other non-metal counterparts found in the oxygen family (group 16 of the Periodic Table): sulfur and tellurium. Its outer electronic configuration is 4*s*^2^4*p*^4^. The atomic number and weight of Se are 34 and 78.96, respectively [173]. Selenium possesses over 20 different isotopes, among which only 6 are stable: ^74^Se, ^76^Se, ^77^Se, ^78^Se, ^80^Se, and ^82^Se [174]. Its melting point is relatively low (~217 °C) and its photoconductivity is high (~8 × 10^4^ S·cm^−1^) [175]. Moreover, selenium shows a catalytic activity toward organic hydration and oxidation reactions, intrinsic chirality, high refractive index, large birefringence, and relatively large piezoelectric, thermoelectric, and nonlinear optical responses [176,177]. In terms of allotropy, selenium can exist in amorphous (a-Se) and crystalline varieties (c-Se): gray (trigonal) selenium (containing Se_n_ helical chain polymers) known as “metallic” selenium; rhombohedral selenium (containing Se_6_ molecules); three deep-red monoclinic forms: α-, β- and γ-selenium (containing Se_8_ molecules); amorphous red selenium, and black vitreous selenium [178]. Crystalline selenium is thermodynamically the most stable structure, exhibiting an atomic radius of 1.17 Å [179]. Several studies indicate that the phase transition between c-Se and a-Se occurs in the charge/discharge process [44,180,181]. a-Se is an efficient photoconversion material, frequently used in several imaging applications, including ultrahigh-sensitivity pickup tubes [182] and solid-state image sensors [183]. Nevertheless, a-Se has a poor spectral response at long wavelengths and requires a high operation voltage [184].

c-Se structure appears as an alternative to a-Se in the photoconversion layer of solid-state image sensors. Crystalline selenium, its most stable form, consists of a long helical chain arranged in a hexagonal array [184]. Several reports detailed the synthesis and properties of c-Se. For instance, Takiguchi et al. fabricated single crystals of trigonal Se with a cylindrical shape and a diameter of about 8 mm [185]. Liu et al. studied the photoconductance of single-crystalline selenium nanotubes (SC-SeNTs) under a 633 nm illumination of various intensities; their results suggest that SC-SeNTs are potentially good photo-sensor materials as well as very effective solar cell materials [186]. Moreover, the synthesis of uniform nanowires of c-Se with uniform lateral dimensions in the range of 10–30 nm was documented [175]; these nanowires can potentially be converted, under adequate conditions, into other functional materials, such as ZnSe and CdSe. Another study assembled c-Se films via doping with different amounts of various halogens, such as chlorine (Cl): 0.50 and 500 ppm; bromine (Br): 50 ppm; and iodine (I): 50 ppm, to investigate the concentration effect on surface enhancement [184]. This technology is suggested to help in the design of super-high-definition imaging systems.

The physicochemical properties of SeNPs have been extensively explored. Chen et al. indicated that in the case of chitosan-stabilized SeNPs the molecular weight of chitosan regulates the Se biological and physicochemical properties, including crystallinity, surface charge density, and hydrophobicity [187]. Zhang et al. proved that chitosan-selenium nanoparticles (CTS-SeNPs), of 80–120 nm in size and different weights, exhibited excellent physicochemical stability after 30 days of storage [92]. Yu et al. showed that CTS-SeNPs, with a particle size smaller than 180 nm, remained stable for 60 days [121]. In addition, Hageman et al. studied the effects of pH (6–9) and temperature (20–50 °C) on the structure, morphology, and stability of biogenic SeNPs using scanning electron microscopy, X-ray diffraction, and light microscopy [188]. As a result, selenium particle crystallinity, shape, and color can be controlled by temperature and pH; for instance, gray crystalline hexagonal acicular SeNPs form at mild temperatures or high pH, whereas red amorphous nanospheres prevail at low temperatures and low pH.

### 4.2. Optoelectronic Properties

Owing to their inherent quantum confinement, SeNPs have distinct, striking shape- and size-dependent physical properties. Selenium is a typical semiconductor with a band gap of 1.6 eV (775 nm) [189]. Due to its ability to absorb X-rays and its high resistivity, ranging from 1012 to 1014 Ω, Se has been considered as an outstanding option for photodetectors and xerographic photoreceptors with ultra-low dark current and high sensitivity [190,191,192,193]. Since selenium is one of the primary substances that possess photoelectric conductivity [57], potential detection applications of a-Se have been investigated, mainly due to its ultra-high photosensitivity, by using avalanche multiplication inside the solid, for example, in imaging photodetectors using low-dose X-rays [194,195], X-ray photoelectron spectroscopy (XPS) and Raman spectroscopy [196], nitrogen (N)-doped diamond cold cathode [197], or driven by a diamond cold cathode [198]. On the other hand, c-Se possesses lower concentrations of selenium than a-Se, thus exhibiting less non-radiative recombination loss. Besides being low cost and highly scalable [199], c-Se has been employed to fabricate solar cells [179,200]. Moreover, Sharma et al. used a genetic algorithm-based code, which consists of universal structure prediction evolutionary xtallography (USPEX) and molecular dynamics, to obtain at least 70 distinct equilibrium geometries for each selenium cluster [201]. The authors analyzed the structural features of Se clusters, including the bond length, bond angle, point symmetry, and shape of the geometries, demonstrating that the lowest energy geometries are one-dimensional rings (buckled or distorted) with each atom possessing only two nearest neighbors.

The optical properties of nanomaterials are highly influenced by multiple factors, such as the size, shape, surface modification, doping, and interactions with other materials [202,203,204]. Unique features, such as the nanoscale dimension, increased energy level spacing (quantum effect), and surface plasmon resonance, determine the size-dependent optical properties, enabling several applications in the biomedical field, energy, catalysis, and environment [174,205]. The NP size distribution can be estimated by optical absorption and luminescence spectra generated by quantum confinement effect [206]. For instance, Rajalakshmi and Arora found that a 0.235 eV blueshift appears in the optical absorption and photoluminescence (PL) energy of SeNPs, which is useful to estimate the particle size [207]. Lesnichaya et al. showed that SeNP polydispersity broadens the absorption and excitation-dependent luminescence spectra [206]. In addition, laser irradiation reduces the size of spherical β-SeNPs (69 nm) below 3 nm and converts them into more closely packed α-Se quantum dots (QDs); then, surface defect density and electron trap level of QDs increase with irradiation time, which decreases energy levels [208]. Another study used optical spectroscopy to show a usual blue shift in the optical spectra of α-monoclinic SeNPs of the order of 40 Å, demonstrating a band gap widening [176]. This blueshift in the band gap energy of Se in comparison with its bulk counterpart appears when the particle size is smaller than its Bohr excitation radius; thus the bandgap is enlarged due to the quantum confinement effect [189]. In this connection, biomolecules, such as proteins and amino acids, absorb light and provide thermodynamic stability [209,210]. Fourier transform infrared (FTIR) analysis suggests that a strong interaction between Se atoms and proteins present in *P. alcaliphila* may be responsible for a drastically decreased intensity of spectral peaks of SeNPs [211]. The optical properties can promote light-induced release of drugs either covalently bonded to or encapsulated with SeNPs [57].

### 4.3. Catalytic Properties

Selenium has been used as a platinum-free, methanol-tolerant cathode material with great stability and electrocatalytic activity, generating chemical resistance to oxidation and hydrolysis [212,213]. In recent years, SeNPs have attracted special attention, particularly due to their unique redox properties, large surface areas, efficient catalytic activity, and low toxicity [214]. Relying on UV–Visible spectroscopy, Dumore and Mukhopadhyay employed the 1-diphenyl-2-picrylhydrazyl free radical scavenging (DPPH-FRS) reaction as a model to monitor the catalytic activity of aqueous selenium nanoparticles (Aq-SeNPs) at different pH (6, 6.5, 7 and buffered 7) [215]. Following pseudo-first-order kinetics, the FRS reaction depended on DPPH concentration since the rate of DPPH-FRS reaction increased proportionally with the amount of Aq-SeNPs, proving that the catalytic reaction occurs at the NP surface. Likewise, other studies have reported on the excellent electrocatalytic performance of selenium-containing compounds, either as a counter-electrode material for dye-sensitized solar cells [216] or as a cathode catalysts for methanol fuel cells [217].

Semiconductor chalcogens, such as those made of selenium, have a direct bandgap, and can be potentially used for the degradation of dyes due to their thermoconductivity, anisotropy, and high photoconductivity [218,219,220,221,222,223,224,225,226,227]. For instance, Ameri et al. described the photocatalytic discoloration of the anionic triphenylmethane dye, bromothymol blue (BTB), using biogenic SeNPs under ultraviolet (UV) illumination (15 W) for 60 min [228]. Another study showed that single-crystalline Se nanorods (SeNRs) were able to degrade methylene blue (MB) in the dark after a short period of irradiation [229]; the superb catalytic performance of SeNRs over commercial nanoparticles was due to the efficient interior charge carrier transfer, and thus the enhanced carrier utilization efficiency. Likewise, Tripathi et al. evaluated the photocatalytic activity of biogenic fluorescent SeNPs in MB decomposition under UV irradiation [230]. Zhang et al. demonstrated the visible light-driven photocatalytic capacity of super-long single-crystalline t-SeNRs for methyl orange (MO) degradation [231]. A similar study reported that monoclinic, spherical SeNPs degraded rhodamine B (RhB) in the presence of H_2_O_2_ more efficiently than t-SeNRs by comparing to results published by another group [232]. This investigation highlights the mechanism of the remaining photomemory effect mechanism of pre-irradiated spherical SeNPs in the dark.

Furthermore, when doped with selenium, bismuth sulfides increased the degradation rate of MB under visible-light irradiation [233]. These improvements were presumably caused by photoelectrons and holes generated by Se dopants in Bi_2_S_3_ photocatalysts. Moreover, Se-doped copper oxide NPs (Se-doped CuO NPs) were used to build a photo-Fenton based degradation system for 4-bromophenol under UV irradiation for 90 min in the presence of H_2_O_2_, achieving a rate of 0.057 min^−1^ [234]. Therefore, doping Se confers extraordinary photo-absorption properties, increases NP surface area, and enhances the in situ generation of hydroxyl radicals. In addition, biosynthesized SeNPs using *W. somnifera* leaf extract exhibit excellent photocatalytic activity in the gradual degradation of MB from deep blue to colorless dye solution under sunlight; besides holes, superoxide and hydroxyl radicals were identified to be involved in this process [160].

SeNPs, obtained using the proper reducing agents, such as ascorbic acid, have proven to be a suitable adsorbent for the removal of copper cations from aqueous solution [235,236]. Further, biogenic SeNPs have been employed as effective and fast adsorbents for zinc ions, mainly through inner-sphere complexation [237]. Another study showed that negatively charged biogenic SeNPs, produced by aerobic granular sludge in a sequencing batch reactor (SBR), efficiently removed Cd(II) [238]. The resulting monolayer maximum adsorption capacity was 59.7 mg g^−1^, enhanced by increasing pH but decreased by increasing adsorbent dosage. In addition to this, selenium combined with ruthenium NPs increased the electrocatalytic oxygen reduction reaction (ORR) by enhancing the oxygen adsorption site and via the electron bridge features of selenium [239].

### 4.4. Biological Properties

As an essential micronutrient, selenium is integrated into 25 selenoproteins in the form of the amino acid selenocysteine (SeCys). In addition, selenium modulates a myriad of key biological processes, such as the cellular response to oxidative stress, cellular differentiation, immune response, redox signaling, and protein folding [75,240]. Moreover, selenium plays important biological roles in maintaining thyroid activity, immunity, and homeostasis through the production of oxidoreductases, such as glutathione peroxidases (GPX), iodothyronine deiodinase (DIO) and thioredoxin reductase (TrxR), and the plasma selenium transport protein (SePP1) [75,80], and preventing several pathologies, such as cancer, diabetes, and aging-related diseases, to name a few [241,242,243]. The main selenoprotein families are GPXs that include (i) five Se-dependent members and other non-Se-dependent GPX isoenzymes, which have oxidoreductase functions and also regulate the immune response; (ii) DIOs that catalyze the conversion of T4 (thyroxine) to T3 (triiodothyronine) and rT3 (reverse T3); and (iii) TrxR, which modulates the transcription and signal transduction functions [244,245] (Table 2). To investigate its biomedical applications, the biological properties of selenium need to be understood; however, they are not yet fully unraveled. Hence, the present critical review outlines some key biological features of SeNPs.

#### 4.4.1. Antioxidant Properties

An antioxidant is a substance that greatly inhibits or delays the oxidation mechanism while the antioxidant activity measures the inhibition rate of the oxidation process [249]. The antioxidant activity of SeNPs is principally associated with the mammalian selenoenzymes GPX, TrxR, and IDO [250]. Selenium, as part of the antioxidant defense system in the liver, plays an essential role against oxidative stress. It has been demonstrated that Se supplementation can enhance enzyme levels, such as GPX, that prevent reactive oxygen species (ROS) accumulation and decrease cellular damage [251,252]. GPXs are able to actively detoxify a variety of peroxides, such as H_2_O_2_, phospholipid and fatty acid hydroperoxides, and hydroperoxyl groups of thymine [174]. TrxRs exhibit a detoxifying action by forming a redox system with its substrate, thioredoxin. These metabolic processes produce the most common free radicals in nature: reactive nitrogen species (RNS) and ROS. ROS derive from oxygen and include peroxyl radical, superoxide radical, perhydroxyl radical, hydroxyl radical, and non-free radical species, such as hydrogen peroxide and singlet oxygen. RNS and ROS are highly unstable, as their outermost electron shell is occupied by an unpaired electron; this leads to the removal of electrons from other compounds to attain stability, which yields a chain reaction cascade that may produce more reactive species.

Research has demonstrated that excessive levels of ROS may cause oxidative stress and redox imbalance in the cell [253]. This can disrupt or damage proteins, DNA, and lipids, resulting in cardiovascular and neurodegenerative diseases, e.g., Parkinson’s and Alzheimer’s [254]. Selenium has attracted attention because its antioxidant properties are predominantly exerted owing to its incorporation into selenoproteins that can catalyze the reduction of disulfide bonds in proteins and peptides [255,256]. Indeed, Se is the main component of the antioxidant enzymes glutathione peroxidases (GPXs), thioredoxin reductases (TrxRs), and iodothyronine deiodinases (DIOs) that protect cells from oxidative stress. For example, selenite, which is an essential dietary supplement for mammals, is present in the active center of the antioxidant enzyme GPX and protects membrane lipids and macromolecules from oxidative stress [257]. Notably, in vitro and in vivo investigations have demonstrated that all selenium compounds under different oxidation states (2−, 0, 4+ and 6+) enhance selenoprotein expression; thus, selenium compounds under different oxidation states have shown great bioavailability as precursors for selenoprotein biosynthesis [89,258]. For instance, Tobe and Mihara found that selenide is involved in synthesis of selenophosphate synthetase (SPS), which consequently produces selenophosphate (SeP), the key selenium donor for the synthesis of selenoproteins and selenium-modified tRNA [259]. Hence, it is of great importance to understand the biotransformation of selenium and reaction mechanisms of the enzymes implicated in selenium metabolism.

SeNPs have proven efficient in enhancing the activity of selenoenzymes to combat oxidative stress with equal effectiveness and less toxicity when compared to MeSeCys, SeMet, and selenite [73]. SeNPs possess radical scavenging properties and reduce oxidants, including 1,1-diphenyl-2-picrylhydrazyl (DPPH), superoxide anion (O_2_^•−^), singlet oxygen (^1^O_2_), and carbon-centered free radicals [42,260,261,262]. This function is size-dependent since smaller SeNPs possess a higher free radical scavenging potential. Moreover, SeNPs are shown to restore T3, T4, glutathione (GSH), superoxide dismutase (SOD), and catalase levels in animal models, and decrease K_2_Cr_2_O_7_-induced oxidative stress in thyroid glands [73,103].

#### 4.4.2. Scavenging Mechanism of Reactive Oxygen Species

ROS are chemically reactive molecules, produced through a myriad of extra- and intra-cellular pathways, that include at least one oxygen atom in each molecule [263]. They include free radicals, a species containing one or more unpaired electrons of oxygen, such as superoxide (O_2_^•−^), hydroxyl radical (^•^OH), and singlet oxygen (^1^O_2_), as well as nonradical oxidizing agents, such as hypochlorous acid (HOCl) and hydrogen peroxide (H_2_O_2_), formed by the partial reduction of oxygen and singlet oxygen [263]. Mitochondria are the main intracellular source of O_2_^•−^, produced by a side reaction of the respiratory chain. The superoxide anions are formed through the conversion of a small percentage of oxygen molecules (1–2%) that are not reduced to water in the mitochondrial electron transport chain (ETC) [263].

The ability of selenium compounds to scavenge ROS is well documented [51,257,263]. For instance, the dose-dependent free radical scavenging activity (FRS) of water-soluble SeNPs (Aq-SeNPs) using 1,1-diphenyl-2-picrylhydrazyl (DPPH) and 2,2′-azinobis (3-ethylbenzothiazoline-6-sulphonic acid) (ABTS) is revealed by a gradual color change of DPPH from intense purple to light yellow, and of ABTS radical from bluish-green to colorless in the presence of Aq-SeNPs at slightly acidic to neutral pH (6.0, 6.5, 7.0 and buffered 7.0) [215]. Importantly, the scavenging ability of the NPs was found to be stronger than that of sodium selenite. Furthermore, SeNPs of 103 nm in size, stabilized with chitosan of different molecular weights (CTS-SeNPs), exhibited a capability to scavenge free radicals at different levels of DPPH, ABTS, and lipid peroxide models [261]. The efficient penetration of CTS-SeNPs into cells and tissues prevents the accumulation of ROS and lipofuscin (LF), protects GPX activity, and decreases selenium in vitro and in vivo cytotoxicity. In addition, SeNPs of different sizes, ranging from 5 to 200 nm, possess important effects both on scavenging free radicals and protecting DNA from oxidation in a size-dependent fashion; as found in an in vitro model, the smaller, the better [261].

**Figure 4:**
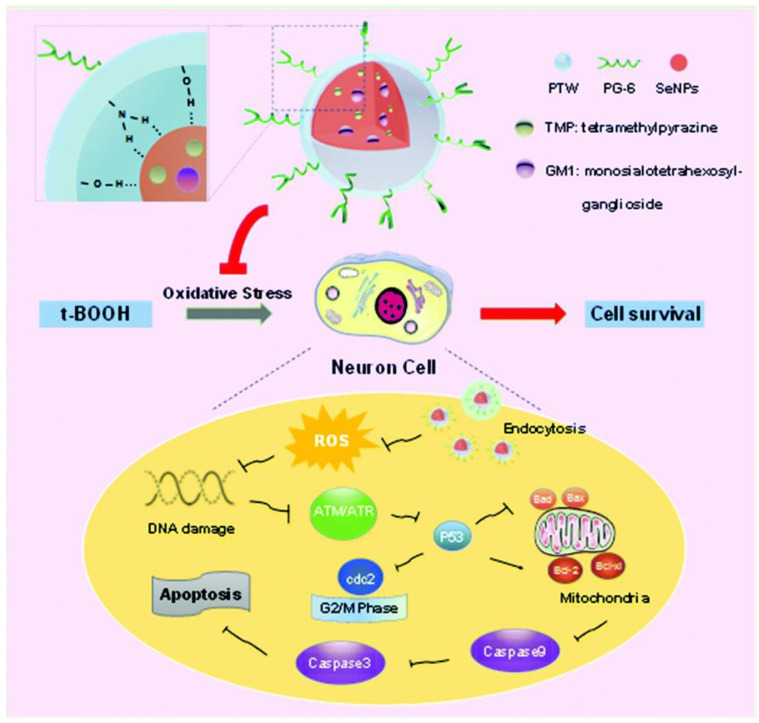
Structure of SeNPs@GM1/TMP and their protective activity against t-BOOH induced neuron cell death. Reproduced with permission from Ref. [264]. 2019, the Royal Society of Chemistry.

Neuroprotective drugs in tandem with ROS scavenging nanocarriers constitute excellent agents to synergistically protect neurons and help restore nerve function. For instance, multifunctional SeNPs were modified with the soluble polysaccharide–protein complex (PTW) and PG-6 peptide (PLGLAG) and loaded with the therapeutic agents monosialotetrahexosylganglioside (GM1) and tetramethylpyrazine (TMP) to effectively treat spinal cord injury (SCI) (Figure 4) [264]. These SeNPs@GM1/TMP were found to attenuate ROS overproduction, prevent mitochondrial dysfunction by up-regulating the expression of pro-apoptotic proteins Bcl-2 (B-cell lymphoma-2) and Bcl-xl (B-cell lymphoma-extra-large), down-regulating the expression of anti-apoptotic proteins Bax (Bcl-2 associated X) and Bad (Bcl-2 associated agonist of cell death), and inhibit the activation of p53 and mitogen-activated protein kinase (MAPK) pathways. They also display protective effects against tert-butyl hydroperoxide (t-BOOH)-induced G2/M phase arrest and apoptosis. Behavioral assessments in mice demonstrated that SeNPs@GM1/TMP constitute promising therapeutic agents to potentially improve the function recovery of rats after SCI.

#### 4.4.3. Pro-Oxidant Activity

Selenium exerts dual effects: at low concentrations, it possesses antioxidant activity by maintaining the intracellular redox status, whereas at higher concentrations, it acts as a pro-oxidant that generates oxygen radicals and provokes apoptotic cell death [248]. Among all the explored inorganic nanoparticles, much attention has been placed on SeNPs due to their cytotoxic activity since ROS are generated inside malignant cells [265,266]. Furthermore, SeNPs are found to be potential tools in fighting drug resistance, either as effective chemotherapeutic agents or as excellent carriers for gene and drug delivery [174]. A study showed that ROS generation by SeNPs is the initial major cellular event prior to cell cycle arrest and/or apoptosis [267]. Indeed, SeNPs enter malignant cells via receptor mediated endocytosis, mainly due to an acidic pH state with redox imbalance [268,269,270,271,272]. This process leads to an NP pro-oxidant behavior by free radical generation, on one side, which induces mitochondrial membrane disruption and, consequently, the leakage of mitochondrial proteins and endoplasmic reticulum (ER) stress on the other side [61,273,274,275]. Hence, several apoptotic molecular pathways which are regulated by SeNPs are activated or modulated, including TLR4/TRAF3/MFN1 (toll-like receptor-4/TNF receptor associated factor 3/mitofusin-1) [276]; p53, MAPK (mitogen-activated protein kinases) and AKT (protein kinase B) [277,278,279]; Bcl-2 family proteins [280]; ROS/JNK (c-Jun N-terminal kinase) [281]; PI3/Akt/mTOR (mammalian target of rapamycin); mTOR and NF-κB (nuclear factor kappa B) [282]; and caspase apoptotic pathways [268,280,283]. The regulation of these pathways is crucial for oncogenic signaling due to a considerable decrease in cellular proliferation and angiogenic signaling by obstructing the growth-promoting signaling in the vicinity of tumor cells. For instance, Pi et al. reported that SeNPs significantly reduce the adhesion force and Young’s modulus of MCF-7 cells, leading to a diminished expression of trans-membrane CD44 molecules and necrosis of MCF-7 cells [284]. Zeebaree et al. demonstrated that spherical biogenic SeNPs, synthesized using *Asteriscus graveolens* leaves, enhance the level of ROS and lipid peroxidation while causing the apoptosis of HepG2 by glutathione depletion and a decrease in the mitochondrial membrane potential [285].

#### 4.4.4. Production of Reactive Oxygen Species

ROS have been recognized as signal mediators implicated in cell growth, differentiation, cycle progression, and death [286]. In regards to oxidative stress, ROS overaccumulation in cells leads to a reaction with different cellular components, causing oxidative cellular injury and cell death [287]. The term “oxidative stress” is attributed to the perturbations of the physiological redox homeostasis when the rate of cellular reduction is exceeded by the rate of cellular oxidation [248]. When ROS overwhelm the cellular antioxidant defense system, either through enhanced ROS levels or a decreased cellular antioxidant capacity, oxidative stress occurs. Particularly, selenium compounds possess a high capacity of exerting oxidative stress by oxidizing thiols and generating ROS, thereby termed as redox-active selenium compounds, e.g., selenocysteine, selenite, methyl selenic acid, and MeSeCys [89]. This clearly demonstrates that selenium does not only have antioxidant properties, but also pro-oxidant properties. Thus, wherever applicable, redox-active selenium compounds are not antioxidant by themselves, but only when supplied at dietary dose levels equivalent to physiological optima and incorporated into selenoproteins with oxidoreductase functions. On the other hand, at supra-physiological levels, redox-active selenium compounds can induce oxidative stress, becoming a novel tool in cancer therapy based on ROS-mediated mechanisms [288]. The effectiveness of selenium compounds for in vivo chemoprevention relies on their capability to regulate the cell cycle, stimulate apoptosis, and restrain tumor cell migration and invasion in vitro [90,289].

**Figure 5:**
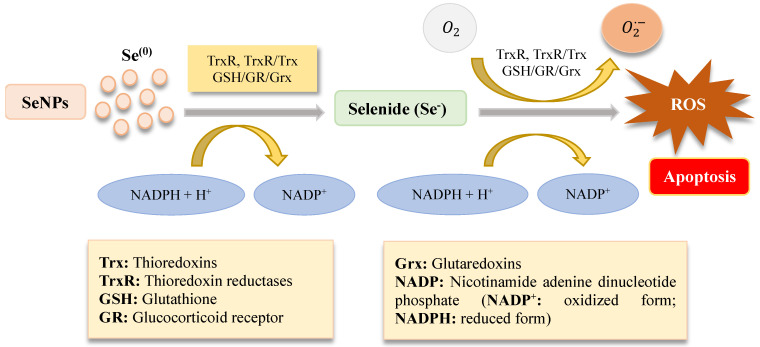
The mechanism of ROS generation mediated by SeNPs. Adapted with permission from Ref. [174]. 2021, The Royal Society of Chemistry.

SeNPs upregulate the activity of selenoenzymes with more efficiency and less toxicity when compared to other selenocompounds, thereby serving as a potential antioxidant and chemopreventive agent [58,96,290]. SeNPs can be reduced into selenide by a thioredoxin (Trx)- or glutaredoxin (Grx)-coupled glutathione system to produce ROS more efficiently than selenite, especially at low levels of NADPH. This is because elemental selenium requires only a single step reduction to selenide anion, thus triggering redox cycling with oxygen [291]. This process leads to a rapid and selective hyper-accumulation of SeNPs in cancer cells, which causes catastrophic oxidative stress and cell death. This underlying concept relies on two observations: firstly, the presence of higher basal levels of ROS in cancer cells compared to normal cells; secondly, cancer cells possess lower tolerance to increased levels of ROS than normal cells [288,292,293]. The process of ROS production, mediated by SeNPs, is illustrated in Figure 5.

The intraperitoneal delivery of SeNPs has emerged as an effective and safe approach in preventing the growth of cancer cells in the peritoneal cavity without remarkable liver toxicity symptoms [294]. For example, Zhao et al. demonstrated that SeNPs delivered to hepatocarcinoma-22 cells in the peritoneal cavity of mice induce ROS production and cause protein degradation and apoptotic response [293]. This study showed that GSH can stimulate a dose-dependent redox biotransformation of SeNPs to generate ROS in a pure enzymatic system, especially given that GSH is the cell’s most abundant thiol-containing small molecule. Another study indicated that a minimal concentration of 2 μg·mL^−1^ of biogenic SeNPs inhibits the proliferation of prostate adenocarcinoma cell line, PC-3, by a ROS-mediated activation of necroptosis [295]. Furthermore, Y. Wang et al. reported on the inverse relationship between SeNPs size and ROS production mediated by a GSH system encompassing GSH, GSH reductase (GR), and NADPH [296]. They found that the smaller SeNPs (35 nm) were more active than larger SeNPs (91 nm) in inhibiting in vitro and in vivo cancer cell accumulation through an ROS mediated mechanism [296].

The process of Se-induced apoptosis is associated with Se chemical species and their metabolism, altering some cellular morphologies including nuclear breakdown, chromatin condensation, membrane blebbing, cell rounding, and formation of apoptotic bodies that are eliminated via phagocytosis [257]. It is well-known that apoptotic cascades can originate from intrinsic mitochondrial, extrinsic receptor, or endoplasmic reticulum (ER) stress-mediated signaling pathways [79]. Although different mechanisms are proposed to explain the key role of Se in the cell cycle and apoptosis, the complete process is complex and not yet fully understood. It is correlated with the chemical forms and doses of selenium, and encompasses the activation of caspases, protein kinase signaling, p53 phosphorylation, and ROS generation [79,255,293,297]. It is also known that selenium compounds possess caspase modulation activity, causing programmed cell death. For instance, Se-containing heterocycles, such as 1,2-[Bis(1,2-benzisoselenazolone-3-(2*H*)-ketone)] ethane (BBSKE), fosters the activity of caspase 3 against tongue cancer Tca8113 cell line [298]; 2,5-Bis(5-hydroxymethyl-2-selenienyl)-3-hydroxymethyl-*N*-methylpyrrole (D-501036) increases the activity of caspases 3 and 9 [299]; and methyl selenic acid activates caspases 8 and 9 in combination with tamoxifen in both tamoxifen-sensitive and tamoxifen-resistant breast cancer cells [289].

The effect of selenium compounds on caspases, also known as cysteine-aspartic-specific proteases, includes the fragmentation of inter-nucleosomal DNA and the induction of the mitochondrial-dependent/independent apoptosis pathway [79]. The intrinsic mitochondrial pathway is the main process for apoptotic caspase activation in mammals, especially owing to the mitochondrial release of cytochrome c (Cyt-c) that creates an apoptosome complex through the oligomerization with Apaf-1 and procaspase-9 [79]. For example, the Se-containing polysaccharide SeGLP-2B-1 disrupts the mitochondrial membrane potential and enhances the cytosolic Cyt-c levels and the activity of caspases 9 and 3 [300,301].

### 4.5. Virucidal Activity

Nanobiotechnology has enabled the design of smart molecular diagnosis/treatment approaches for viral infections [302,303,304,305]. For example, synthetic NPs exhibit high antiviral activity and can closely mimic the virus and strongly interact with its virulent proteins due to morphological similarities [306]. In addition, nanostructures can deliver viral antigens in a controlled manner, activate follicular dendritic cells or B cells, antigen cross-presentation, as well as induce humoral/cellular immune responses [307].

SeNPs are found to possess much lower acute toxicity and similar or higher bioavailability when compared to other Se species, such as selenite [308,309,310,311], selenate [312], selenium dioxide [313], Se-yeast [314], selenomethionine [290,315], and methylselenocysteine [47]. In addition, SeNPs have significant antiviral, antibacterial, antiparasitic, and cytotoxic activity [54,316,317,318]. For example, SeNPs decorated with amantadine were able to reverse drug resistance caused by the H1N1 influenza virus infection through the inhibition of caspase-3 activity and suppression of the neuraminidase activity [319]. A similar study demonstrated the superior antiviral capability of *β*-thujaplicin-decorated SeNPs against H1N1 via the regulation of AKT and p53 signaling pathways [320].

At the onset of the COVID-19 pandemic, selenium was proposed as a weapon of choice to fight against SARS-CoV-2 [321,322,323,324,325,326,327]. For instance, Jin et al. reported that a synthetic redox-active selenium compound, ebselen, is a strong inhibitor of the main SARS-CoV-2 protease that enables viral maturation within the host [328]. This study suggested that high Se intake might hypothetically inhibit SARS-CoV-2 proteases and promote a higher cure rate. Knowing that selenium deficiency is linked to severe virulence, intravenous Se therapy and high-dose selenite pharmaconutrition have been proposed to be effective at reducing the occurrence and the progression of acute respiratory distress syndrome (ARDS), multiorgan failure, and new infections in COVID-19 patients [329]. Overall, human Se levels are crucial in antioxidant, anti-inflammatory, and immune effects in COVID-19 patients; thus, it is important to study the impact of Se excess and deficiency in mitigating COVID-19 symptoms, especially in patients with pre-existing comorbidities or long-term diseases [330].

**Figure 6:**
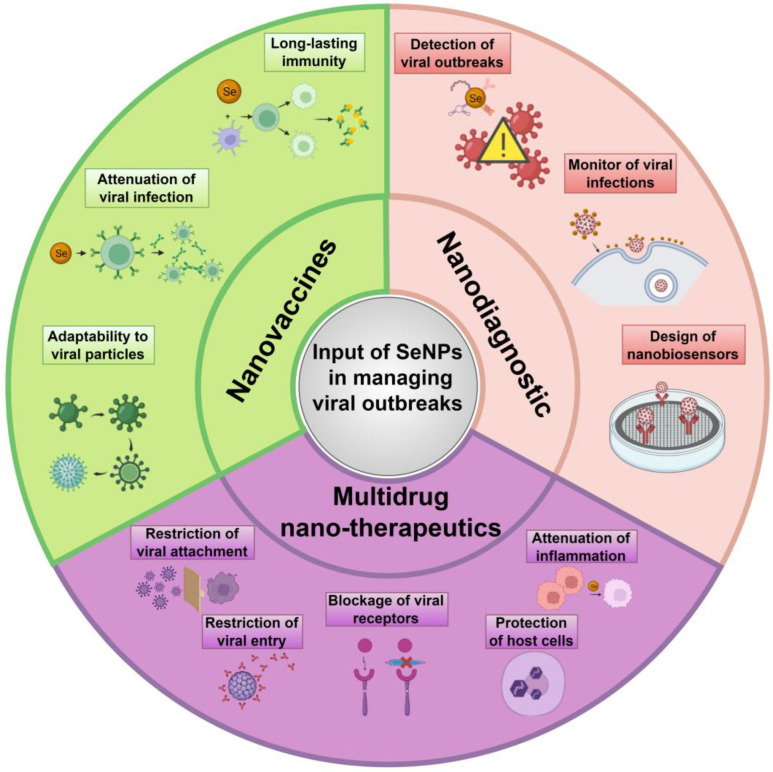
Biomedical application of SeNPs against viral infections.

A unique experimental study used lateral flow immunoassay kits (LFIA) relying on SeNPs modified with SARS-CoV-2 nucleoproteins for the combined detection of anti-SARS-CoV-2 IgM and IgG in human sera, and succeeded in exhibiting recent SARS-CoV-2 infection within just 10 min detectable by the naked eye [331]. In addition, the sensitivity and specificity of the kits were clinically examined with real-time polymerase chain reaction (RT-PCR) tests in COVID-19-diagnosed patients and non-infected controls, amounting to 93.33% and 97.34%, respectively. Finally, there were no cross-reactions with rheumatoid factor and positive sera for influenza A, influenza B, and antinuclear antibodies. Similar studies have also designed point-of-care systems based on SeNPs to detect IgG and IgM against SARS-CoV-2 [332,333]. Although little research has been conducted to highlight their potential in mitigating and controlling COVID-19 pandemic, SeNPs clearly constitute superior detection tools and antiviral nanotherapeutics amenable to containing and/or combating viral outbreaks and pandemics. Figure 6 summarizes the biomedical role of SeNPs in diagnosing and curing viral infections.

## 5. Pharmacokinetics and Cellular Interactions of Selenium Nanoparticles

Nanomedicine is increasingly offering novel nanoparticle-based technologies for therapy and diagnosis [72,334,335,336]. The extraordinary properties of nanomaterials provide a safe and efficient basis for personalized medicine, which promotes tailored therapies considering the patient’s specific characteristics for the best response and highest safety margin [337,338]. In vitro and in vivo pharmacokinetics, i.e., absorption, distribution, metabolism, excretion, and toxicity (ADME-Tox) studies have been the rule for pharmaceutical organic drugs. Likewise, nanomaterials should be subjected to the same in vitro and in vivo ADME-Tox studies [24,339]. NPs need to reach the targeted organ or tissue to accomplish the desired action with both efficacy and safety. Therefore, nanotherapeutics rely on effective NP cellular uptake and tissue or tumor permeability that both depend on various factors, such as the size, shape, and surface chemistry of the NPs, as well as the biological (micro)environment, the specific location, and the targeted tissue [340,341,342].

### 5.1. Interaction of Selenium Nanoparticles with Cells and Their Components

**Figure 7:**
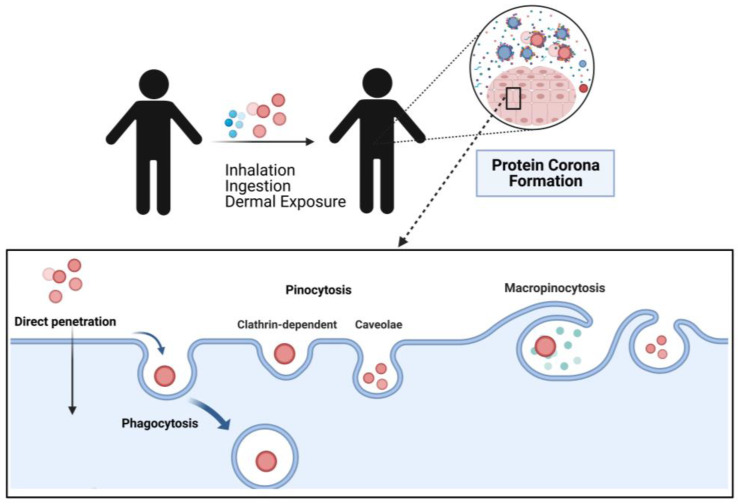
Schematic representation of the different manners through which NPs enter the human body and are internalized inside the cells.

The interaction of nanomaterials with cells and lipid bilayers is crucial in several biomedical applications, such as drug and gene delivery [280,343,344,345], diagnostics, phototherapy, and imaging [34,219,346,347,348]. Besides interacting with biological entities (i.e., organs and tissues), NPs cross the cellular barriers and are internalized by cells through endocytosis to accumulate in targeted organs and are eventually cleared [349]. The cell internalization of NPs occurs via several pathways, such as direct penetration, phagocytosis, and pinocytosis [350]. On the other hand, the body internalizes the NPs via inhalation, ingestion, and dermal exposure, to name a few, as depicted in Figure 7 [351]. Although this particular capacity leads to beneficial therapeutic applications, some potential adverse effects regarding NP toxicity have been observed [351]. In consequence, the investigation of the NP interactions with their microenvironment, mainly with other nanomaterials and biomolecules, is crucial to determine the efficacy of nanoscale materials [341,349,352].

Several factors determine the success of the NP uptake and interaction with cells, including the intrinsic NP physicochemical properties, such as the shape, size, coating and morphology, and crystalline structure, in addition to the biological environment characteristics and the transformation of NPs during the test, e.g., transformations owing to the formation and adsorption of a protein layer, known as protein corona [353,354]. The size and shape of the NPs directly affect their cellular uptake rate, which is also related to their residence time in the circulatory system [337]. The NP shape plays a special role in their internalization as it impacts their interaction with the cells. In addition, the NP symmetry controls their trajectory within the body since hydrodynamic forces regulate their transport [355]. For example, several studies concur that spheres are the most effective in terms of cellular uptake due mainly to their isotropic shape, which allows a constant distribution of acting forces and a tendency to remain in the blood flow longer [356,357]. Furthermore, spherical NPs must overcome a minimal membrane bending energy barrier, when compared to their non-spherical counterparts.

The importance of NP-cell interactions has been acknowledged by several authors [352,358,359,360]. However, little research has addressed the quantitative analysis of NP–cell interactions, which is essential to fully understand nano-bio mechanisms and nanotoxicology of cell-surface bound and intracellular NPs [361]. Lately, novel analytical mass spectrometry (MS)-based methods, such as laser ablation inductively coupled plasma mass spectroscopy (LA-ICP-MS), time-resolved ICP-MS (TR-ICP-MS), nano secondary ionization mass spectrometry (nano-SIMS), and mass cytometry that fundamentally combines flow cytometry with time of flight mass spectrometry (ToF-MS), have been introduced [353]. Several quantification studies on the interaction of NPs and cells under different experimental conditions (i.e., type of NPs and cell lines) using SC-ICP-MS for a diversity of applications, such as nanotoxicity, drug delivery research, and optimization of techniques for the green synthesis of NPs, have been developed [362,363,364,365,366]. For instance, Hu et al. quantified intracellular amounts of SeNP uptake by γδT cells using ICP-MS and found that the total uptake amount was ~9 × 10^−5^ μmol of SeNPs per million of cells [97].

The analysis of NP uptake and biodistribution has gained much importance in recent years, mainly to evaluate effective concentrations of clinically administered NPs. According to the literature, the most remarkable biomolecules interacting with the NP surface are nucleic acids and proteins [367,368]. Nucleic acids are convenient receptors for molecular nano-structures, demonstrating potent synergistic activity due to their mechanical rigidity, physicochemical stability, and high specificity of base pairing [369]. On the other hand, proteins possess various binding sites owing to post-translational modifications in addition to specific and non-specific adsorption capability. All these interactions confer stability, sustained enzymatic activity, and immune-biocompatibility to nanomaterials [370,371,372,373].

The main factors that influence the coupling of NPs with biomolecules or other NPs are known as the interaction drivers, and include van der Waals forces, electrostatic or magnetic interactions, and molecular forces, based on complementarity between nanomaterials, their distance, and their geometry [374]. Furthermore, the interaction between surface molecules greatly depends on the surface functionalization with one or different chemical reactive moieties [375]. For instance, NPs in biological milieus are surrounded by interacting biomolecules that are able to change or saturate their surface, resulting in surface coating modification that, in turn, may alter their unique properties, initially designed functionalities, and desired targeting capabilities, in addition to altering their cytotoxicity and influencing their pharmacokinetic features and accumulation [376,377,378,379].

**Figure 8:**
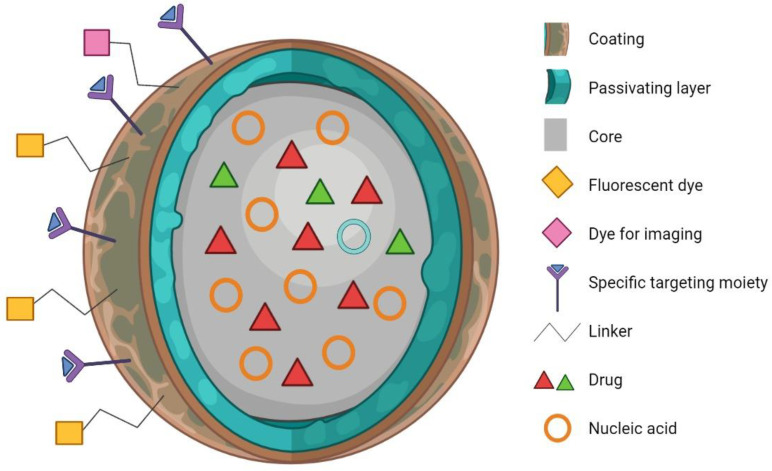
Schematic representation of multifunctional NPs that may include an imaging component, a targeting element, and a therapeutic constituent.

Surface modification of NPs using functional molecules/particles/polymers can increase their cellular interactions and uptake by tuning their overall properties to fit the targeted applications [380]. Multifunctional NPs have various and different interactions with biomolecules, and are embedded within human proximal fluids inside cells and culture media [337,369]. To achieve this multifunctionality, NPs are bioconjugated with several entities that may include the diagnostic imaging domain, the targeting ligand, and therapeutic moiety to yield multifunctional formulations consisting of therapeutic-loaded NPs, also known as theranostic NPs (Figure 8) [381,382,383]. This constitutes a key step forward in nanomedicine towards personalized medicine with promising applications in drug delivery, cancer treatment, and diagnosis, among others [8,24,337].

For instance, monodisperse and homogeneous spherical SeNPs have been successfully modified with a dinuclear luminescent Ru(II) complex resulting in a multifunctional nanocarrier-based delivery system (NDS) that delivers the siRNA targeting tumor-MDR1 gene in cisplatin resistant adenocarcinomic human alveolar basal epithelial cells (A549 cells) [384]. Another study presented amine-terminated generation 5 polyamidoamine (PAMAM) dendrimers (G5.NH_2_)-modified SeNPs (G5@SeNPs) for the systemic dual delivery of MDR1 siRNA and cisplatin to down-regulate P-glycoprotein and reverse multidrug resistance [385]. Through gel retardation assay, cellular uptake, and transfection studies, these multifunctional G5@SeNPs are found to enhance siRNA loading, release efficiency, and gene-silencing efficacy. In addition, siRNA- and polyethylenimine (PEI)-modified SeNPs are shown to improve the apoptosis of HepG2 cells [386]. Therefore, all these studies demonstrate that multifunctional SeNPs are effective nanosystems for chemotherapy and gene therapy technology.

When NPs interact with plasma proteins, a protein corona forms on their surface. This has been widely studied due to its significant effects on therapeutic NPs [387,388,389,390]. Indeed, it can considerably modify the NP shape, size, surface charge distribution, and susceptibility to aggregation. Its formation also dictates the subsequent biological fate of NPs within the body [342,359] and modulates various biological behaviors, such as cell-uptake, toxicity and immunogenicity [391]. Since this layer is important in the NP-cell interactions, various studies have investigated the parameters affecting the adsorption of proteins on the NP surface in physiological fluids and the role played by the corona in the mechanism of NP uptake by the cells [392]. As a result, the formation of an individualized protein corona might be useful for targeted therapy and, consequently, for personalized medicine approaches [393].

Chakraborty et al. studied the formation of coronas, consisting of serum most abundant proteins, i.e., human serum albumin (HSA), IgG, and transferrin, to encapsulate SeNPs that had been previously functionalized using one of the following surfactants: cetyltriammonium bromide (CTAB, cationic), sodium dodecyl sulfate (SDS, anionic), and Brij-58 (non-ionic) [394]. As a result, HSA was found to increase the antioxidant capacity of SeNPs, whereas the presence of IgG and transferrin reduced their radical scavenging activity. Moreover, the protein corona formation over functionalized SeNPs enhanced their size and decreased their cellular uptake and subsequent toxicity, except for transferrin-coronated NPs that showed increased uptake and cytotoxicity. In addition, protein coronation is significantly influenced by NP functionalization [395]. In fact, protein quantification and densitometry studies showed that cationic SeNPs (CTAB-SeNPs) promote maximum corona formation and possess higher affinity towards the predominantly negative surface potential of serum albumin. This suggests that binding factors, such as electrostatic forces, attachment via cysteine, hydrogen bonding, and entropy-driven binding, govern the process of protein coronation [395]. Moreover, the study of molecular interactions between biogenic SeNPs, synthesized using yeast extract, and HSA by employing a microwave plasma optical emission spectrometry operating in a single-particle mode documented that the potential biomedical application of SeNPs greatly depends on their surface functionalization and capability to form a protein corona [396].

Polysaccharides (PS) have been widely used as functionalizing agents for nanomaterials, especially due to their unique properties, such as excellent biocompatibility, stability, and biodegradability [397,398,399]. In addition, they increase the residence time of PS-functionalized NPs at the target site and the permeation/bioavailability of loaded drugs or biomolecules [400], enhance the specific interaction with biological targets [401], and improve cell-permeability and cancer-targeting ability [398]. For example, SeNPs decorated with mushroom PS–protein complexes (PSP) achieved remarkably enhanced cellular uptake via endocytosis which, in turn, improved their antiproliferative activity [49]. The capping with PSP was carried out through strong physical adsorption of PS hydroxyl groups and protein imino groups to yield PSP–SeNPs, whose main target organelles are lysosomes on MCF-7 human breast carcinoma cells. Moreover, *Spirulina* PS (SPS) from the food-grade blue-green microalga *Spirulina platensis* are known to have an essential biological role in free radical scavenging, DNA repair, immunostimulation, and antiviral effect [402]. Thus, SPS could be employed as a surface decorator of NPs to improve their cell-penetrating capabilities, prolong their circulation time, and prevent plasma protein adsorption. For instance, SPS-functionalization of monodisperse spherical SeNPs enhances their cellular uptake capability and cytotoxicity against various human cancer cell lines, including A375 melanoma cells [403]. A comprehensive survey of Se-carbohydrate combinations including PS-decorated SeNPs is provided by Li et al. [404].

### 5.2. Key Role of Selenoproteins in the Pharmacological Activity of SeNPs

As mentioned earlier, selenium is a unique trace element which presents a pharmacological behavior by its incorporation into selenoproteins. Several selenoproteins are essential enzymes that include at least one SeCys in their active sites to exert catalytic and antioxidant activities [405,406]. Selenoproteins play several physiological roles [250,407,408,409,410], such as regulating the immune functions [408], protecting against oxidative stress [411], preventing cardiovascular disorders [412,413], regulating thyroid hormone metabolism [414,415], influencing the occurrence of type 2 diabetes [416], protecting against cartilage redox homeostasis and the progression of arthropathies [417], chemoprevention and chemotherapy [406], modulating energy metabolism in neural cells [418], and enhancing male fertility [419]. However, there is little information on the effect of SeNPs on the pharmacokinetics and pharmacodynamics of selenoproteins [73]. For example, SeNPs have a better effect on glutathione S-transferase (GST) activity when compared to selenoproteins regardless of supra-nutritional or toxic levels [290]. Moreover, SeNPs are used to design a safe and effective strategy for a highly therapeutic efficacy of cytokine-induced killer (CIK)-based cancer immunotherapy [35]. Since the safety profile of nanomaterials to a large extent determines their biomedical applications, no induced hemolysis is noticed when SeNPs are incubated with human blood, highlighting their hemocompatibility. In addition, SeNPs are gradually metabolized into selenocysteine (SeCys_2_), which subsequently regulates the expression of multiple selenoproteins and other metabolites in CIK and cancer cells [35]. This unique strategy enables a CIK-SeNPs co-treatment that induces specific immune responses against tumor progression via the production of natural killer cells and the priming of tumor-associated macrophages. All these findings are helpful in translational medicine towards the development of efficient treatments for diseases associated with Se metabolism [32]. Additionally, SeNPs improve selenium bioavailability and facilitate selenoprotein expression when selenium level is low [127]. Indeed, the encapsulation of selenium in chitosan networks (CTS-SeNPs) likely increases its retention and/or delivery to induce selenoprotein expression and prevent Se-induced damage to DNA.

### 5.3. Pharmacokinetics of Selenium Nanoparticles

Pharmacokinetics investigates the scale and rate of ADME-Tox of drugs in the body through precise and rigorous experimental methods [24,420]. By nanosizing its formulation, the drug dissolution rate can be increased to promote improved absorption and bioavailability [421,422]. Therefore, NPs are useful to deliver drugs and enhance tissue selectivity due to their selective uptake in specific tissues [24,341,422]. Moreover, nanocarriers alter the pharmacokinetic properties of drugs by enhancing their effectiveness and diminishing their adverse effects [423]. The pharmacokinetic profiles of the parent drug and the drug associated with NPs are often different [424]. Thus, studying the pharmacokinetics and biodistribution of drugs formulated as NPs is essential to comprehend and predict their effectiveness and side effects. For delivery purposes, an optimal theranostic NP model should display suitable release kinetics of the drug in specific concentrations at the target site [24].

The physicochemical properties of NPs are essential for pharmacokinetic modulation as they dictate the immediate pharmacological response in the body following their administration [425]. For instance, the shape, size, surface chemistry (PEGylation, ligand conjugation), surface charge, and composition influence the pharmacokinetics, intracellular penetration, and tumor bioavailability [341]. Moreover, NPs prolong the half-life of drugs in blood circulation, decrease their apparent volume of distribution, and significantly reduce their degradation and clearance. Depending on the method of preparation and the desired therapeutic effect, the drug can be dissolved, adsorbed, attached, entrapped, or encapsulated within NPs [426,427]. Regardless of their compositions, all NPs must possess a reasonable half-life in the blood circulation, selective targetability, and efficacious clearance from the body after the drug delivery to target tissues [428,429,430,431,432]. To obtain the adequate NP pharmacokinetic features for clinical applications, it is required to regulate their hydrodynamic diameter, shape, and surface properties. The general process includes: (1) systemic circulation and reticuloendothelial system (RES) interaction, (2) extravasation and tumor penetration, and (3) interaction with target cells [433]. The specific pharmacokinetic parameters include the volume of distribution, half-life, mean residence time, maximum concentration, bioavailability, permeability, clearance, and area under the time–concentration curve [434].

In the case of selenium, Se metabolic cycling and excretion from the human body include both inorganic and organic molecular species of Se present at different oxidation states including 2−, 2+, 4+ and 6+ [435]. Nevertheless, little research has been conducted to show the formation of elemental or metallic Se (Se^0^) as part of these metabolic processes [435]. For instance, MeSeCys, an organic Se compound with potential anticancer activity, is found to be an advantageous supplementation of Se due to its lowest toxicity among all the selenocompounds while still being greatly bioavailable [86]. The same study also showed that the positive effects of various selenocompounds on the activity of GPX1 and on the generation of the glycoprotein selenoprotein P (SEPP) do not correlate with their toxicity levels but rather with molecule-specific properties [86]. Further, MeSeCys has minor sub-chronic oral toxicity and no genotoxicity at doses far above the daily nutritional Se level (0.5, 0.7, 0.9 mg per kg of body weight) after 90-day oral exposure [436]. Although the pharmacokinetics of sodium selenite has been investigated in various animal models, the biosafety dose of Se is still unclear due to the narrow safe dose range of Se and the distinct animal physiological and pathological conditions [437]. For instance, selenium-enriched yeast (SeY) had higher bioavailability in rats than sodium selenite, while plasma-free SeMet was found to be the ideal biomarker of SeY status in vivo [438]. Furthermore, rapid absorption and slow excretion of sodium selenate in the blood of healthy piglets were observed; this pharmacokinetic process conforms to the two-chamber open model [439]. Additionally, variations in antioxidant systems in piglets as a function of Se levels were noticed, thereby providing a more complete understanding of risk assessment and clinical application of Se supplementation. On the other hand, the safe dose level of intravenously administered sodium selenite, defined as maximum tolerated dose (MTD), was reported to be 10.2 mg·m^−2^ in terminal cancer patients and implied the importance of kidney function in the excretion of Se from selenite since the pharmacokinetic results demonstrated a linear increase in plasma Se concentration with respect to total Se dose [440]. Lastly, no apparent adverse effects of high dose of repeated selenite administration on physiological selenium homeostasis were reported [440].

The pharmacokinetic and pharmacodynamic profiles of two high doses of parenteral selenite in patients with systemic inflammatory response syndrome (SIRS) showed that the maximum glutathione peroxidase activity appeared only at very high dose [441]. As a result, the very high dose of 2000 µg (25.30 µmol) of selenium supplied as selenous acid by short-term bolus injection, followed by a continuous daily intravenous infusion of 1600 µg (or 20.24 µmol) for 10 days, was very effective in replenishing serum selenium to physiological levels and safely maximizing the antioxidant activity of the selenoenzyme GPX3. Furthermore, it has been shown that three selenium compounds (sodium selenite, methyl selenocysteine, and seleno-1-methionine) were well-tolerated and assessed safe to be used at 400 µg elemental selenium per day in a study population of twenty-four cancer patients, revealing negligible genotoxicity and minor reductions in lymphocyte counts [442].

Although the above therapeutic applications of Se compounds by conventional introduction may seem effective, the delivery of nano-formulated Se has distinct pharmacokinetic advantages such as specific drug delivery, controlled release, high metabolic stability, high membrane permeability, enhanced bioavailability, long duration of action, less toxicity, and therapeutic efficacy [50,61,74,76,90,443]. SeNPs are convenient for administration since they can be delivered by various routes including oral and intravenous ones [444]. At the nanoscale, selenium is also used to improve the pharmacokinetic properties of drugs. For instance, chitosan-decorated SeNPs constitute an excellent carrier of the therapeutic peptide BAY 55-9837 for type 2 diabetes mellitus by decreasing its renal clearance rate [445]. In addition, insulin-loaded SeNPs (Ins-SeNPs), fabricated using ionic cross-linking/in situ reduction, exhibit enhanced antidiabetic effect through a controlled insulin delivery and an outstanding stability in the digestive fluids [446]. Based on ex vivo intestinal imaging and cellular internalization, the transepithelial transport ability of SeNPs to overcome the absorption barrier was assessed, and it was found that Ins-SeNPs not only get into the cytoplasm, but also enter the nuclei. Finally, Ins-SeNPs exhibit excellent hypoglycemic effects after oral administration, requiring a lower oral dose to achieve a long-acting glycemic reduction than insulin alone for the same time and concentration (0.75 μg·mL^−1^) [446].

In addition, Arg-Gly-Asp (RGD) peptide-decorated and doxorubicin-loaded selenium nanoparticles (RGD-SeNPs) targeting tumor vasculature significantly enhanced the cellular uptake and antiangiogenic activity of SeNPs in vitro and in vivo on human umbilical vein endothelial cells (HUVEC) [447]. The as-designed nanosystem led to bioresponsive triggered drug doxorubicin (DOX) release by disassembly under acidic conditions with the presence of lysozymes and cell lysate [447]. Another study reported on the hybridization of SeNPs with niosomes as lipid nanoparticles (NISM-B@SeNPs) to open a new approach in drug delivery for cancer treatment studies that potentially exhibit good in vivo biocompatibility [343]. Moreover, Se-functionalized liposomes (SeLPs) were developed as a DOX delivery vehicle to prolong the systemic circulation of liposomes by in situ selenium coating and enhance the anticancer effect via the synergy between DOX and Se [339]. A dual-loaded nanocarrier system of the antiretroviral drug Etravirine (TMC-125) and SeNPs was fabricated to evaluate the NP biodistribution in potential human immunodeficiency virus (HIV) reservoirs in vivo in Sprague Dawley rats [448]. The in vivo pharmacokinetic study showed the controlled release potential of the nanocarrier along with high stability, prolonged on-target residence time, low clearance, and a higher accumulation of the dual-loaded nanocarrier in remote HIV reservoir organs such as the brain, ovary, and lymph node.

A nanoselenium-coating biomimetic cytomembrane nanoplatform (BMMP) was prepared as a drug nanocarrier by using *P. geniculate* for manganese and doxorubicin codelivery and mitochondrial targeted chemotherapy [449]. The pharmacokinetic studies revealed that approximately 50% of BMMP-Mn^2+^/Se/DOX was retained in the body at 8 h post-injection, implying that BMMP-Mn^2+^/Se/DOX had a long blood circulation time. In addition, the nanoplatform exhibited excellent biosafety and exerted long-acting effects on tumors, being completely excreted from the body at 96 h post-injection with no obvious side effects from its residue [449]. Se-coated nanostructured lipid carriers (SeNLCs) of around 160 nm were developed for improving the oral bioavailability and the curative effect of berberine, an antidiabetic phytomedicine [450]. The results showed that the berberine-loaded SeNLCs (BB-SeNLCs) had an entrapment efficiency of 90% and greatly enhanced the oral bioavailability of berberine, which was approximately 6.63-fold higher than that of berberine solution. From the abovementioned cases, it is evident that SeNPs are emerging as valid pharmacological tools for further in vitro and in vivo studies on conjugates with drugs, drug candidates, targeting agents, and molecular probes.

## 6. Green Nanotechnology: A Better Avenue for SeNP Bioapplications

Despite tremendous advances in the application of nanotechnology in the diagnosis and treatment of different diseases, several challenges are yet to be adequately addressed, such as bio- and cyto-compatibility, as well as selectivity and efficiency of NPs [451]. The conventional synthesis of nanomaterials often involves the use and/or the generation of toxic/harmful reagents and substances (i.e., solvents, catalysts, and reducing and capping agents), which affect the environment and patient response. Moreover, bringing nanomaterials from the laboratory to clinical or industrial applications has been slow and challenging due to the poor understanding of the new potential hazards introduced by nanotechnology and the absence of suitable policies to manage emerging risks [452,453,454,455]. Hence, bionanotechnology may hold the solution and constitute a sustainable alternative by offering better and safer processing methods for NP production [65,171,456,457,458,459,460,461]. This growing approach aims at exploring the capabilities of natural, widespread, and renewable resources as part of the starting reagents in the NP production process to eliminate or, at least, reduce the NP hazards to the environment and human health, and, ultimately, to substitute the existing toxic reagents/products with new environmentally friendly products that are benign, eco-friendly, sustainable, biocompatible, and safe [462,463]. Indeed, organic, non-toxic stabilizing agents lead to controlled sizes and shapes, tailored biological responses (e.g., cytotoxicity, inflammation), and enhanced biodistribution of biogenic NPs [451].

Green nanomaterials have demonstrated potential applications in medicine as anticancer, antidiabetic and antioxidant agents, as well as for bio-sensing purposes [464]. For instance, green drug delivery nanosystems display efficient targeted recognition and controlled release, high biocompatibility, and decreased toxicity [465]. Thus, this has triggered a sustained demand for green nanotechnology-driven drug delivery systems fueled by significant developments of diverse delivery devices, such as inorganic NPs, quantum dots, polymeric NPs, dendrimers, nanostructured lipid carriers, solid lipid NPs, etc. [466]. Further, the rise of green nanomaterials is accompanied by sustainable, low-energy, and low-cost procedures for the manufacturing of different tissues that diminish the consumption of toxic materials [467].

Green approaches produce highly stable and biocompatible SeNPs that increase efficiency and minimize side effects [155,468]. Indeed, some metabolites present in plant extracts, such as polyphenols, saponins, vitamins, carbohydrates (including polysaccharides), flavonoids, alkaloids and tannins, are excellent reducing and capping agents of SeNPs that make them safer, more stable [469], and suitable for several potential applications in biomedical sciences [42,60,61,62,63,470]. A recent survey comprehensively addresses the biosynthesis of SeNPs using a variety of plant extracts relating the properties of the synthesized nanostructures (composition, size, shape, stability) with the conditions used for this green route (temperature, time, Se precursor, and extract concentration) [471]. Since biosynthesized SeNPs are less polydisperse and do not aggregate under physiological conditions, they have emerged as effective tools in medical and pharmaceutical sciences to treat different diseases [61,472]. In addition, eco-friendly metallic NPs are safely translated in medicine and serve as safe nanotheranostic agents/platforms [468,473,474]. However, some important concerns need to be considered prior to the use of SeNPs in clinical translational applications, such as the safety profile, pharmacokinetics and pharmacodynamics, and specificity and sensitivity in the biological milieu.

## 7. Biomedical Applications of Biogenic Selenium Nanoparticles

SeNPs are bioactive entities that might be easily made biologically available to play a crucial role in many oxidoreductive processes [91]. In addition, SeNPs possess a regulative effect to support the correct functioning of the body and offer outstanding health benefits to treat/cure various diseases [32,58,475,476]. The following sections detail the use of biogenic SeNPs for different therapeutic purposes, including anticancer, antimicrobial, and anti-diabetic activities, in addition to gene and drug delivery.

### 7.1. Antioxidant Activity

Antioxidants are compounds that prevent the generation of free radicals as well as scavenge them when produced during various biochemical reactions in animals and plants, therefore playing a significant role in protecting against oxidative stress and neurodegenerative and cardiovascular diseases [58,477,478]. The antioxidant potential of biogenic NPs relies on the redox potential of phenolic and flavonoid compounds present at their surface [128,479,480]. Since selenium is implicated in antioxidant defense systems and significantly contributes to maintaining the redox homeostasis [481,482,483,484], SeNPs display a protective activity against cellular damage [167,485,486]. To date, an overwhelming number of studies indicate that SeNPs possess great antioxidant ability and free radical scavenging efficiency that potentially protect tissues and cells from oxidative damage [160]. Moreover, SeNPs exhibit antioxidant activity with less toxic effects than zero-valent selenium (Se^0^) [51] or sodium selenite [155]. In addition, biogenic SeNPs exhibit a higher antioxidant activity with less toxicity to healthy cells than selenium dioxide [260]. It has also been proved that SeNPs, fabricated using *Theobroma cacao* L. bean shell extract as the reducing and capping agent, are highly stable and possess a better antioxidant activity than the extract itself [487]. Similar behaviors of SeNPs, fabricated using different natural extracts, have been reported [160,488,489,490]. However, *Ephedra aphylla* extract displays a better antioxidant activity than SeNPs produced using the same extract owing, most likely, to the presence in the extract of phenolics, flavonoids, and tannins in greater amounts [491].

Biogenic SeNPs exhibit numerous biological properties. For instance, SeNPs of 5–200 nm can directly scavenge free radicals in vitro [492]. In addition, their size has a considerable effect on their antioxidant properties since smaller NPs are more efficient in capturing free radicals [262]. This was corroborated by other studies that also established the dependency of antioxidant activity on particle concentration [109,490,493]. Additionally, the capping moieties, such as quercetin and gallic acid, play an important role in the antioxidant potential of ecofriendly SeNPs [494], and of bimetallic Ag-Se NPs [495]. Table 3 summarizes the antioxidant activity of biogenic SeNPs, obtained via different methods.

The most used in vitro technique to measure the free radical scavenging activity (RSA) of SeNPs is 2,2-diphenyl-1-picrylhydrazyl (DPPH) as it is simple, rapid, facile, sensitive, and stable [249]. It is based on the reduction of methanolic DPPH solution by donating an electron or hydrogen atom to form a non-radical, stable, and pale yellow/colorless molecule: 2,2-diphenyl-1-hydrazine [316,490].

Several studies explored the strong antioxidant activity of biogenic SeNPs synthesized by *Lactobacillus casei* in oxidative stress-caused intestinal epithelial barrier dysfunction [496,497,498]. As a result, SeNPs can alleviate ROS mediated mitochondrial dysfunction via Nrf2-mediated signaling pathway, increase the number of goblet cells, reduce the production of ROS, increase GPX activity, and preserve the mitochondrial functions. Therefore, SeNPs could be employed to treat oxidative stress-related intestinal disorders. Similarly, biogenic SeNPs, mainly coated by polysaccharides, exhibit antioxidative and anti-inflammatory effects in protecting intestinal epithelial cells against H_2_O_2_ and ETEC K88-caused injury, and maintaining the intestinal epithelial barrier integrity [499]. This amount of work has led to a consensus stating that biogenic SeNPs present significant antioxidant activity and may serve as a potential antioxidant supplement or ingredient [155,488] and neuroprotective agent [500].

### 7.2. Antimicrobial Activity

The antimicrobial capability of SeNPs stems from their large surface to volume ratio, which allows them to set better contact with microorganisms, thus leading to improved antimicrobial activity [147]. Therefore, SeNPs can be used in several fields including infectious control, surface treatment of biomedical instruments, pharmaceutical industry, cosmetics, and food manufacturing [155].

#### 7.2.1. Antibacterial Activity

The antibacterial activity of selenium compounds is attributed to the generation of free radicals, including Se oxyanions [501]. These novel active products might constitute a solution to the emerging drug-resistant microorganisms that are considered to be a great current health concern [62,502,503,504]. The unique antibacterial effect of biosynthesized SeNPs has been extensively explored on the basis of morphological and structural changes in the bacterial cells [160,316,490,491,495]. For instance, phytofabricated SeNPs using the aqueous fruit extract of *Emblica officinalis* were found to possess antimicrobial activity against both Gram-positive and -negative bacteria and fungi [155]. The minimal inhibitory (MIC) and bactericidal (MBC) concentrations were 9.16 ± 0.76 and 19.83 ± 1.25 μg·mL^−1^, respectively, against *S. aureus*, and 59.83 ± 2.56 and 97.50 ± 3.27 μg·mL^−1^, respectively, against *E. coli*. A similar study provides the MIC of manufactured SeNPs using aqueous extract of fermented lupin against *Acinetobacter calcoaceticus* (2.343 μg·mL^−1^) and *S. aureus* (1.171 μg·mL^−1^) [149]. In addition, the activity of the same SeNPs against the fungus *Aspergillus* was strain-selective, as they were effective only against *A. flavus* while no activity was shown against *A. niger*.

The NP antimicrobial activity is size-dependent since tiny NPs can easily cross the cell wall and membrane and provoke cell lysis [93,114,155]. For instance, 221.1 nm SeNPs, fabricated using *S. maltophilia*, exhibit a strong antimicrobial activity with an effective concentration (EC_50_) of 26.32 mg·L^−1^ against *E. coli*, 7.59 mg·L^−1^ against *S. aureus*, and 62.37 mg·L^−1^ against *P. aeruginosa* [505]. In addition, the large surface area, small size, and spherical shape are probably responsible for the good antimicrobial activity of SeNPs fabricated using the supernatant of *Lysinibacillus* sp. against *E. coli* and *S. aureus* [68]. Moreover, the anti-biofilm activity of different concentrations (0–2 mg·mL^−1^) of the same biogenic SeNPs against the strong-biofilm producer *P. aeruginosa* was highlighted.

**Figure 9:**
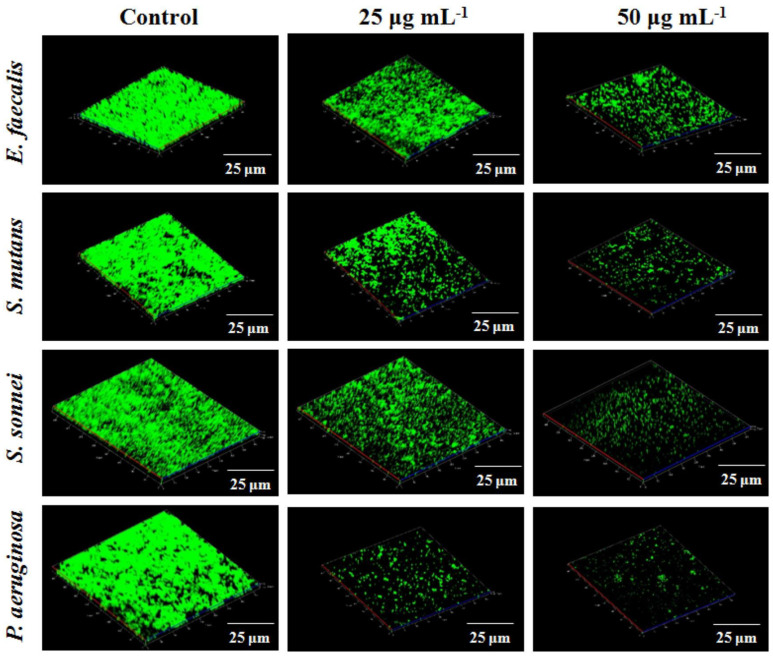
Confocal laser scanning microscopy images of antibiofilm activity of biogenic SeNPs, produced using the aqueous extract of *Murraya koenigii* against Gram-positive (*E. faecalis* and *S. mutans*) and Gram-negative (*S. sonnei* and *P. aeruginosa*) bacteria. Reproduced with permission from Ref. [109]. 2019, Elsevier.

Besides the dimensions, other important features, such as the elemental structure (purity) and shape of SeNPs, should be considered when studying their antibacterial activity [155]. For example, the antimicrobial and antibiofilm ability of SeNPs, manufactured using *S. maltophilia* SeITE02, against different pathogenic bacteria seems to be strictly linked to their organic surrounding cap [62]. This characteristic was tested by exposing NPs to progressively stronger protocols that denature their external organic coating; this resulted in increased MIC values with progressive denaturation. Moreover, SeNPs synthesized using *C. bulbosa* tuber aqueous extract were found to promote inhibitory effects on the growth of certain clinical pathogens, such as *B. subtilis* and *E. coli*, as well as a strong larvicidal activity against the dengue vector, *Aedes albopictus*, with 250 μg·mL^−1^ as the maximum lethal concentration [506]. In this study, the mortality rate of *A. albopictus* larvae is caused by the adhesion and penetration of SeNPs across cell membranes to further hinder the function of membrane proteins. On the other hand, biofilm inhibition assays using biogenic SeNPs resulted in a gradual decline of biofilm thickness at 25 μg mL^−1^ (80–70% reduction) and 50 μg·mL^−1^ (extreme reduction), thus inhibiting any further bacterial proliferation (Figure 9) [109]. Additionally, SeNPs, derived from *B. licheniformis*, inhibit *S. aureus* adherence and microcolony formation on polystyrene, glass, and catheter surfaces [507]. These findings are corroborated by another study showing that SeNPs, fabricated by the whole cell lysate of *Bacillus* sp., inhibited the biofilm formation by *S. aureus*, *P. aeruginosa*, and *P. mirabilis* by 12.42%, 34.30%, and 53.40%, respectively.

Owing to their interactions with DNA and proteins, biogenic SeNPs totally inhibit *S. aureus* growth at a 300 μM concentration within 24 h [508]. Spherical SeNPs of 40 to 120 nm in size, fabricated with the non-pathogenic bacterium *Ralstonia eutropha*, were found to almost totally inhibit (99%) the growth of *P. aeruginosa*, *S. aureus*, *E. coli*, and *Streptococcus pyogenes* at different concentrations ranging from 10 to 300 μg·mL^−1^ [55]. In contrast, the growth inhibition of the pathogenic fungus *A. clavatus* requires 500 μg·mL^−1^ of the same SeNPs. Moreover, the antimicrobial activity of SeNPs, synthesized by *Zingiber officinale* root extract, against Gram-positive (*S. aureus*) and -negative (*E. coli*, *Klebsiella* sp., *Pseudomonas* sp., *Serratia* sp., and *Proteus* sp.) bacteria resulted in MIC values of 150–500 μg·mL^−1^ [501]. On the other hand, SeNPs, produced using *E. coli*, *P. aeruginosa*, *S. aureus*, and methicillin-resistant *S. aureus* (MRSA), are shown to inhibit the bacterial growth by, most likely, altering the bacterial growth cycle by impacting the synthesis of RNA, enzymes, and/or other molecules involved in the cell division [133]. In addition, these SeNPs may cause a systemic failure of the bacterial metabolism leading to cell death owing to induced ROS generation. Similar results are obtained with biogenic SeNPs, synthesized via a plant-mediated process. This is the case, for instance, of SeNPs produced using *Cinnamomum zeylanicum* bark extract, which show a biocidal activity against several bacterial foodborne pathogens (*E. coli*, *Salmonella typhimurium*, *S. aureus*, and *Listeria monocytogenes*), and display a potential edible coating basement [509]. Lastly, interesting antibacterial activities are also obtained using SeNPs fabricated by the aqueous extract of cow urine [510].

#### 7.2.2. Antifungal Activity

Several investigations have reported the antifungal activity of biogenic SeNPs. For example, mycosynthesized spherical SeNPs show an antifungal activity against *Pyricularia grisea*, *Colletotrichum capsica*, and *Alternaria solani*, in addition to the inhibition of the sporulation of *P. infestans* [147]. Moreover, SeNP-enriched probiotics (*L. plantarum* and *L. johnsonii*) combined with extracellular metabolites inhibit the growth of the potent urogenital pathogen *C. albicans* [511]. Additionally, selenium dioxide in culture supernatants enhances the production of soluble metabolites involved in killing the same yeast. Furthermore, the size and crystallinity of chitosan-stabilized SeNPs greatly influence their synergistic antifungal effect against *C. albicans* biofilms in a dose–response manner [113].

SeNPs, produced using the fusarium *T. harzianum*, exhibit excellent antifungal activity and, more specifically, a dramatic deactivation of several synthetic genes (FUM1, PA, TRI5, and TRI6) and toxins of the fungi *Alternaria* (83% TeA and 79% AOH reduction) and *Fusarium* (63% FB1 and 76% DON reduction), opening them new avenues as bifunctional nanomaterials for the biocontrol of phytopathogens and mycotoxins in agriculture and food safety [512]. On the other hand, SeNPs, fabricated using the plant extract of *E. aphylla*, display potent antimicrobial activity against several bacterial and fungal species with an enhanced inhibition zone diameter ranging from 19 to 39 mm [491]. In addition, biosynthesized SeNPs using the leaves of *Diospyros montana* show the highest inhibition zone against *A. niger* (12 mm) when compared to the tested bacterial species using a disc diffusion method at different concentrations (10, 20, 30 and 40 μg·mL^−1^) [490].

### 7.3. Antiparasitic Activity

Biogenic SeNPs show effective and accurate prophylactic effects on acute toxoplasmosis, thereby offering a potential alternative to pyrimethamine and sulfadiazine, whose treatment presents serious side effects [513]. In addition to their strong larvicidal activity against the dengue vector mosquito, *A. albopictus* [506], biogenic SeNPs also exert in vitro and in vivo antiparasitic activity against *Leishmania major*, rendering these nano-objects into novel therapeutic agents for the treatment of localized lesions typical of cutaneous leishmaniasis [514].

### 7.4. Anticancer Activity

Cancer remains a major global health challenge and embraces nearly 100 types characterized by an uncontrolled division of abnormal cells with the ability to metastasize to other parts of the body [515]. Over the previous decades, nanotechnology has gained much importance as a revolutionary approach to combat cancer owing to the unique properties of NPs, such as their size, shape, large surface-to-volume ratio, tunable surface chemistry, and the ability to encapsulate/carry and deliver various drugs, that confer to them many advantages over their bulk counterparts [516]. A fuller understanding of nano–bio interactions should lead to safer and more efficacious nanotherapeutics by overcoming the physiological barriers posed by the tumor microenvironment, which will eventually facilitate the corresponding clinical developments [517]. On the other hand, green nanomaterials are currently intensively screened for the treatment and diagnosis of cancer owing to their high biocompatibility and effectiveness, among which biogenic SeNPs hold great promise [518,519,520]. Building on previously published data [85], Table 4 provides an update and indicates the anticancer activity displayed by a number of biogenic SeNPs against different cancer cell lines.

**Figure 10:**
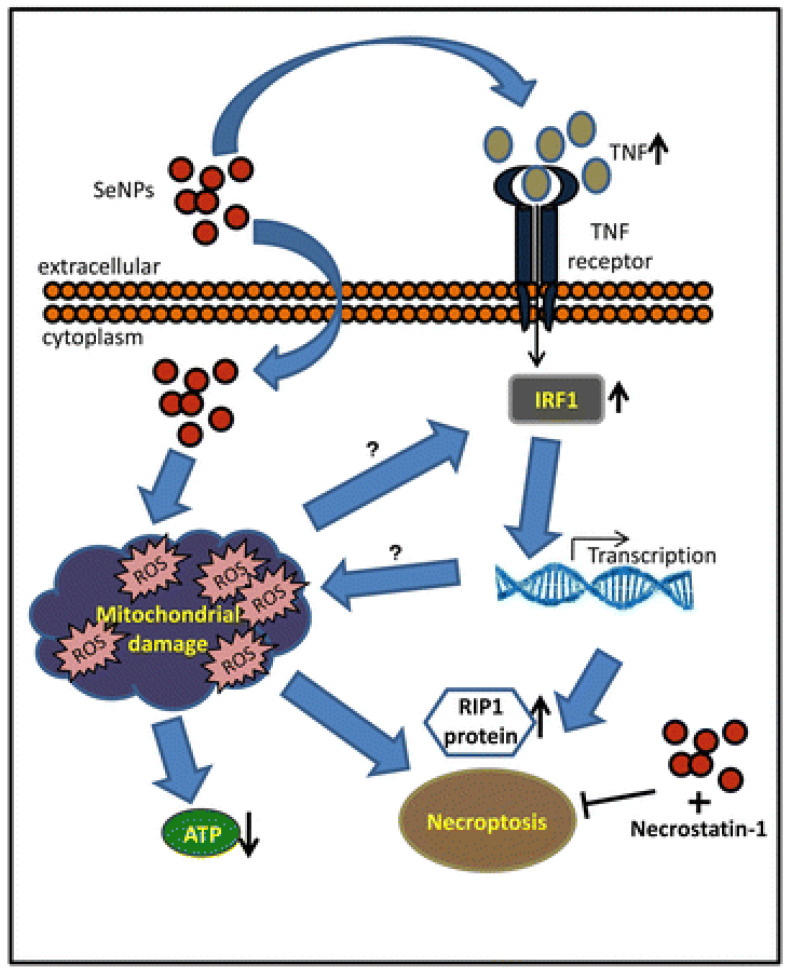
Anticancer effect of biogenic, spherical SeNPs, synthesized by a living culture of *B. lichenisformis*, against PC3 cells via a necroptosis pathway. Adapted with permission from Ref. [295] under the terms of the Creative Commons CC BY license. 2017, Springer Nature.

For instance, biogenic SeNPs are better anticancer, nontoxic, and biocompatible operators than selenite and selenate compounds [50]. Moreover, *B. licheniformis*-derived SeNPs emerge as the safest form of selenium supplementation with potent necroptosis activity against LNCaP-FGC cancer cells, without affecting red blood cell integrity [524]. Similar results were obtained against the PC3 prostate adenocarcinoma cell line (Figure 10) [295]. *Streptomyces bikiniensis*-derived SeNPs demonstrate excellent in vitro anticancer effect against Hep-G2 and MCF-7 malignant cells through a hypothetical mechanism consisting of the increased mobilization of endogenous copper (possibly chromatin-bound copper) of cancer cells and the subsequent pro-oxidant action [521]. Another proposed anticancer mechanism of biogenic SeNPs fabricated using *Idiomarina* sp. PR58-8 is based on the activation of apoptotic pathways by an increased expression of pro-caspase 3 [526]. This is corroborated by the expression of poly(ADP-ribose) polymerase (PARP), and the cleavage of PARP since the activated form of caspase 3 catalyzes the cleavage of pro-PARP, consistent with the role of caspases, a family of intracellular proteases essential in the initiation and execution of apoptosis or programmed cell death through proteolytic cleavage [536]. A similar study reports that biosynthesized SeNPs in combination with X-rays are involved in caspase 3 activation and downstream targets that inhibit the proliferation of lung cancer cells with high cytotoxic effect [523].

**Figure 11:**
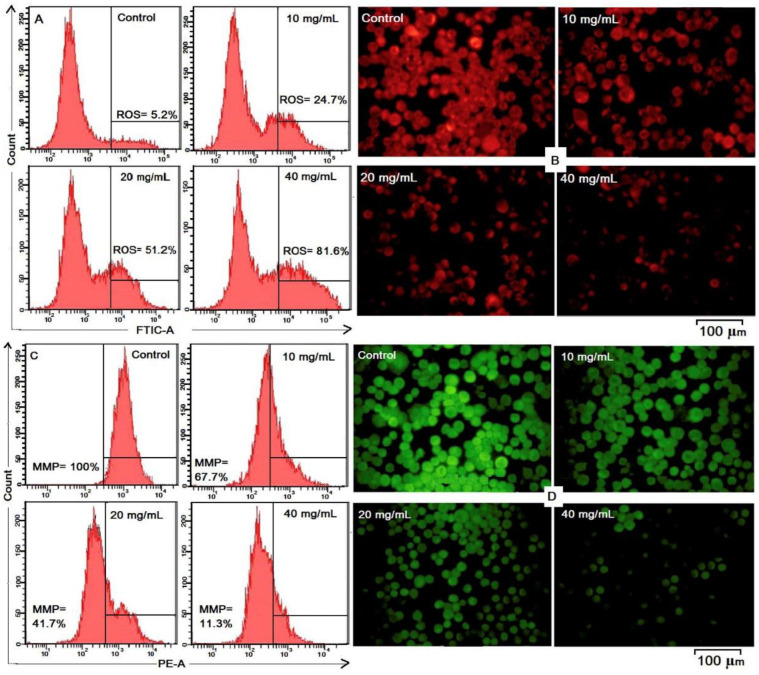
Measurements of (**A**) ROS generation and (**C**) MMP induced by various concentrations (10–40 µg·mL^−1^) of SeNPs using flow cytometry; (**B**,**D**) microscopy illustration of ROS and MMP production, respectively. Reproduced with permission from Ref. [285]. 2020, Elsevier.

Biogenic SeNPs exert their anticancer activity in a dose-dependent manner [491,525,537] and seem to be less cytotoxic to non-malignant cells when compared to their analogs obtained via chemical routes [522], while their surface functionalization has no impact on their anticancer activity nor on the induction of cell cycle arrest [531]. Besides the cytotoxicity pathways discussed above, Se-induced cytotoxicity against malignant cells might also be the result of ROS generation, as in the case of injection of biogenic SeNPs into the abdominal cavity of mice following their inoculation with highly malignant H22 hepatocarcinoma cells [294]. This is corroborated by another study where biogenic spherical SeNPs, synthesized using *Asteriscus graveolens* leaf extract, induce ROS overproduction and mitochondrial membrane potential (MMP) disruption, thus evidencing their high antitumor activity against HepG2 cells through the activation of apoptosis pathways (Figure 11) [285]. Via another mechanism, biogenic SeNPs, produced using hawthorn fruit extract, trigger increased apoptosis rates in HepG2 cells by up-regulating caspase 9 and down-regulating Bcl-2 [529].

Overall, nanostructured Se compounds have significant potential in the fight against cancer due to their chemopreventive activity, antioxidant/pro-oxidant activity as modulators of ROS suppression/production, capacity to modulate inflammatory processes, apoptosis, capacity to inhibit cancer metastasis, selective targeting of tumors over healthy adjacent tissue, and, last but not least, capacity to inhibit multidrug efflux pumps and thereby counteract tumor resistance to established chemotherapeutic agents and inhibit cancer metastasis. These emerging insights concerning Se-based small molecules formulated as NPs and quantum dots open the way towards new anticancer therapeutics, or for adjuvants designed to overcome drug resistance associated with current chemotherapeutic protocols used routinely in clinical oncology [538].

### 7.5. Protective Role of Selenium Nanoparticles against Drug-Induced Toxicity

Notwithstanding the widespread application of chemotherapeutic drugs in clinical tumor treatment, serious toxicity, dose-dependent side effects, and non-specific targeting restrict their therapeutic efficacy [539]. Thus, SeNPs, alone or in formulation, have been considered as a potent agent preventing adverse chemotherapy due to their high bioavailability and low toxicity [90]. For example, SeNPs induce a significant tumor cell apoptosis and an impressive enhancement of the therapeutic effect of irinotecan by a selective modulation of Nrf2-ARE pathway in tumor and healthy tissues [444]. Another report demonstrates that SeNPs, at daily doses of 1 mg per kg body weight, efficiently alleviate bone toxicity caused by the intake of anastrozole—a breast cancer drug—therefore preventing the occurrence of osteoporosis in ovariectomized female SD rats [540]. This study also highlights a process of ossification and mineralization in the femurs of SeNP-treated groups, which probably can be explained by the NP antioxidant and protective action. In addition, biogenic SeNPs protect against adverse effects of antibiotics. For instance, SeNPs, produced using the bacterium *Pantoea agglomerans*, exhibit a protective role in immunological and oxidative stress generated by enrofloxacin (EFX), a broad-spectrum antibiotic, in broiler chickens at a dose of 0.6 mg kg^−1^ of feed [541]. Furthermore, SeNPs display protective effects against gentamicin-induced nephro- and hemo-toxicity in female Swiss albino mice [542], and hexavalent chromium-induced thyroid damage in male rats [103].

Cisplatin (CIS) is a commonly used alkylating agent to treat testicular, ovarian, head, and neck cancers, among others [543]. Despite its wide clinical applications, CIS triggers many side effects, including the obstruction of some cellular processes, e.g., DNA replication and transcription by inducing DNA adducts and establishing DNA cross-links [544]. Several articles provide insight on the protective effects of SeNPs against CIS toxicity [385,545,546]. For example, SeNPs possess a strong antioxidant potential to prevent CIS-induced gonadotoxicity [544]. Moreover, SeNPs surface-functionalized by 6-hydroxy-2,5,7,8-tetramethylchroman-2-carboxylic acid (Trolox) (Se@Trolox) block cisplatin-induced ROS accumulation [547]. In addition, SeNPs decorated with amantadine (Se@AM), remarkably, prevent caspase 3 activation and decrease ROS levels to inhibit the ability of the H1N1 influenza virus to infect host cells, thereby overcoming the emergence of drug-resistant viruses [319]. Furthermore, SeNPs display protective effects in the progression of diabetic nephropathy (DN) by increasing the levels of heat shock protein (HSP-70) [548]. Lastly, SeNPs increase the number of neutrophils and prolong their survival duration in healthy sheep, as evidenced through thiobarbituric acid reactive substances (TBARS) assay [549].

Biogenic SeNPs, synthesized using *Terminalia arjuna* leaf extract, show protective and antigenotoxic effects against arsenite (As^3+^)-induced genetic damage in isolated human lymphocytes [102]. Selenium has been recognized as an effective chemo-protectant against the toxicity of cadmium, exposure to which produces high ROS levels, regarded as Cd-induced neurotoxicity and nephrotoxicity [550]. For example, SeNPs, sodium selenite, and yeast-Se diets display different protective proficiency in Cd-induced testicular damage by improving the expression and synthesis of selenoproteins via the regulation of numerous related transcription factors [551]. Another study ascribes the chemoprotective effects of SeNPs against neuro- and nephro-toxicity of subchronic exposure to CdCl_2_, mainly, to the inhibition of lipid peroxidation and regulation of genes encoding numerous detoxifying and antioxidant enzymes [550].

*T. harzianum*-derived SeNPs exhibit high protective effects in infected maize and pear with *Fusarium* and *Alternaria* as the main parasites, suggesting that these NPs may be applicable as bifunctional nanomaterials for biocontrol of phytopathogens and mycotoxins in agriculture and food safety [512]. Furthermore, biologically synthesized SeNPs using the terrestrial actinomycete *S. griseobrunneus*, at 64 μg·mL^−1^, can eliminate, under UV irradiation, 94% of diclofenac through hydroxylation, oxidation, and decarboxylation [228].

### 7.6. Anti-Inflammatory Activity

Biogenic SeNPs, synthesized using *Trachyspermum ammi*, exhibit anti-rheumatic and immunomodulatory properties in arthritic Balb/c mice, as a SeNP treatment reduces paw edema along with decreased lymphocytic cellular infiltration in liver, kidney, and spleen specimens, as well as improved redox state of inflamed synovium [552]. Moreover, SeNPs, dispersed in phytochemical P-coumaric acid, exert an anti-inflammatory activity by modulating catalase, GPX1, and COX-2 gene expression in a rheumatoid arthritis rat model [553]. In addition, biogenic SeNPs, synthesized using *L. casei*, possess strong antioxidant and anti-inflammatory activity to effectively protect human colon epithelial cells against H_2_O_2_-induced injury [498]. SeNPs are a promising anticonvulsant agent due to their potent antioxidant, anti-inflammatory, and neuromodulatory activities against pentylenetetrazole (PTZ)-mediated epileptic seizures in mice hippocampus [554]. Importantly, SeNPs are more effective than sodium selenite in terms of antioxidant and anti-inflammatory activity against induced eimeriosis in the jejunum of mice; therefore, they could be applied for immunoregulation purposes [555]. The potential anti-inflammatory activity of biogenic SeNPs may be ascribed to their down-regulation of pro-inflammatory genes and mediators (e.g., TNF-α, PGE2, TBAR and NOx) and/or to their further antioxidant activity [556]. Additionally, SeNPs ameliorate the health state of rats with streptozotocin-instigated brain oxidative-inflammatory stress and neurobehavioral alterations by regulating the molecular markers of oxidative stress and tissue damage: Nrf2, caspase 3, and parvalbumin proteins [557].

Macrophages play a vital role in chronic inflammatory diseases (CIDs), and thus regulating their activity is crucial in detecting and reducing chronic inflammation [558]. SeNPs, decorated with *Ulva lactuca* polysaccharides, display anti-inflammatory effects, and relieve the symptoms of acute colitis through the inhibition of the hyper-activation of NF-κB in colon tissues and macrophages [559]. Biogenic SeNPs lower the amounts of H_2_O_2_ produced by the pro-inflammatory-activated macrophages in addition to selectively targeting, imaging, and killing pro-inflammatory-activated ones under photodynamic treatment [560]. Further, SeNPs, stabilized by sulfated *Ganoderma lucidum* polysaccharides, inhibit the inflammation caused by over-activated macrophages in Raw 264.7 cells in a dose-dependent manner [561].

### 7.7. Antidiabetic Activity

Diabetes mellitus (DM) is defined as a group of metabolic disorders characterized by the decrease in insulin secretion by pancreatic islet cells leading to high blood glucose levels (hyperglycemia) [562]. Diabetes is classified into two major types: type 1 diabetes mellitus, or insulin-dependent diabetes, and type 2, or non-insulin dependent diabetes. Type 1 diabetes mellitus causes a deficiency of insulin due to autoimmune or genetic disorders, while type 2 diabetes (T2D) generates an insulin resistance or reduced insulin sensitivity as a result of inappropriate diet or lack of physical activity [563]. Advances in nanotechnology, molecular and biomedical imaging tools, and drug delivery systems are offering new opportunities for early diagnosis and monitoring disease progression in patients with type 1 or type 2 diabetes combined with diminished insulin secretion [562].

Several articles have thoroughly explored the association between selenium concentration and diabetes [564,565,566,567,568,569]. Patients with DM are often affected by oxidative stress, requiring more antioxidant species to reduce the oxidative and inflammatory response [570]. Since selenium is known to possess excellent antioxidant and anti-inflammatory effects against DM [571,572], several studies have investigated the pivotal therapeutic role of SeNPs in alleviating most diabetic complications and insulin resistance [53,140,446,573]. Owing to their antidiabetic potency, SeNPs can increment insulin secretion by preserving the pancreatic β cell integrity, repressing oxidative stress, inducing glucose depletion, and inhibiting pancreatic inflammation [574]. For instance, biogenic SeNPs, fabricated using *Hibiscus sabdariffa* (roselle plant) leaf extract, significantly decreased the oxidative stress indicators of testicular tissue in streptozotocin (STZ)-induced diabetic rats, such as nitric oxide and lipid peroxidation [168]. These findings indicate that these nanocrystals hold great promise in attenuating oxidative damage induced by diabetes. Similar research reveals that the administration of SeNPs prepared with *Catathelasma ventricosum* polysaccharides (CVPS-SeNPs), remarkably, ameliorate body weight, blood sugar level, antioxidant enzyme activities, and lipid levels in STZ-induced diabetic mice, highlighting, thus, their dramatic antidiabetic activity [140]. Although a synergistic effect of CVPS-SeNPs and vitamin E regarding this antidiabetic activity is suggested, the underlying mechanism is not yet clearly understood.

CTS-SeNPs conjugated with a novel peptide, consisting of a recombinant pituitary adenylate cyclase-activating polypeptide (PACAP)-derived peptide DBAYL capable of specifically activating the vasoactive intestinal peptide receptor 2, also known as the VPAC2 receptor, that influences glucose-dependent insulin secretion, enhances insulin sensitivity, hyperglycemia, and lipid profiles, thus demonstrating the potential of this assembly to become a long-acting anti-T2D therapeutic [573]. Moreover, chitosan-decorated SeNPs (CTS-SeNPs) are used to treat T2D by prolonging the in vivo half-life of the therapeutic peptide BAY 55-9837 and slowing its renal clearance rate, proving that BAY 55-9837-loaded CTS-SeNPs possess a desirable sustained-release profile and high stability that could enhance the half-life of low-molecular-weight therapeutics by increasing their apparent molecular size [445]. In combination with metformin (MF), CTS-SeNPs are also effective in the treatment of T2D by mitigating the diabetic complications in a better way than a monotherapeutic approach, and considerably restrict the T2D-induced sperm abnormalities, such as reduced sperm motility, diminished levels of sexual hormones, testicular oxidative damage, and steroidogenesis-related genes dysregulation [575]. Other studies detail the antidiabetic effect of CTS-SeNPs as a monotherapy or as part of a combined therapy with drugs able to swiftly decrease blood glucose and insulin levels [576], in addition to nanohybrid systems to treat diabetic wound infection at mild stage [577].

### 7.8. Diagnostic Applications

Nanotechnology has led to the development of various NP formulations for diagnostic applications, thereby revolutionizing treatment strategies of relevant diseases, whose outcomes depend on early and accurate detection, such as cancer [25,336,426,578,579], diabetes [580,581,582], gastrointestinal disorders [583,584,585], infectious diseases [586,587,588,589], and neurodegenerative disorders [590,591,592]. Imaging and point-of-care technologies are two specific fields that could benefit from the utilization of NPs [593,594]. In the case of imaging (vide infra), NPs appear positioned to play a key role in the future of medical diagnostics due to their many advantages over the conventional contrast agents, including high affinity binding, specific molecular targeting abilities, controlled biological clearance pathways, and prolonged residence time, thereby providing a longer time for imaging with even multimodal and stimuli-responsive attributes [595,596,597]. However, nanodiagnostics remain useful in very limited clinical situations due to complex demands on pharmacokinetic activity and clearance [595]. The application of SeNPs is of particular interest as they have high photoconductivity, piezoelectricity, thermoelectricity, and spectral sensitivity properties [210,219,598]. For instance, optical and photoluminescence properties of SeNPs can be exploited in the fabrication of nanosensors and imaging markers, eliminating the requirement for additional fluorescent tags, such as proteins or dyes [219].

#### 7.8.1. Detection, Biosensing and Diagnostics

Before addressing the specific place of nanoscale Se in detection and biosensing, a brief outline of this emerging area is in order. Nanobiosensors have witnessed tremendous developments resulting in innovative and sophisticated devices, due to the ever-increasing demand to efficiently and reliably sense a great variety of molecules at low concentrations with high specificity and selectivity [464,599,600,601,602,603,604]. Compared to bulk materials, nanomaterials possess large surface-to-volume ratios, which enable them to provide a greater surface to anchor biomolecules of interest [605,606,607]. In addition to this feature exploited in achieving a high-density immobilization of bioreceptors, nanobiosensors benefit from the unique NP properties to enhance biological signaling and transduction mechanisms since these nanostructures catalyze the bioreactions, mediate the electron transfer, amplify the mass change, and refine the refractive index changes [599,608,609].

Conventional procedures to immobilize enzymes include physical adsorption, affinity labeling, covalent cross-linking, and entrapment [610,611,612,613,614,615]. However, these methodologies are often multistep procedures and present different shortcomings such as non-specific adsorption, leakage, and/or partial denaturation of immobilized biomolecules [616,617]. Aiming to tackle these challenges, approaches based on nanostructured platforms offer exceptional benefits in enzyme immobilization including high binding capacity, high catalytic activity, long operational and storage stability, high specific surface area for volume-efficient catalysis, low protein unfolding, and minimal mass transport limitations [618,619,620]. Nano-immobilized enzymes can be tailored into diverse sizes and shapes without employing toxic reagents [621].

In the above-described beneficial context, biogenic NPs, including those based on Se, offer significant advantages over their analogs synthesized by conventional methods, such as a better stability of up to several months that leads to simple, rapid, nontoxic, cost-effective, and handy sensing strategies [42,622,623,624,625]. Indeed, different proteins and biomolecules present in the reaction medium bind to the surface on the NPs, preventing their aggregation or flocculation, and conferring to them long-term stability [626,627,628]. Furthermore, green-chemistry techniques can potentially improve biosensing applications, such as transducers or electroactive labels, especially in NP-based electrochemical detection systems [629,630]. Thus, the emerging greener biosensors can be relevant for point-of-care handling due to their biocompatibility [631].

Hydrogen peroxide (H_2_O_2_) has received particular attention as an important analyte for human metabolism because any imbalance between its generation and consumption can damage lysosomal membranes and DNA or induce apoptosis [632,633,634]. Therefore, reliable, accurate, and rapid sensing techniques for cellular peroxide detection are of paramount importance [635]. For instance, biogenic Se nanorods (SeNRs), fabricated using citric acid and flavonoids from lemon juice, serve as an H_2_O_2_ spectrometric sensor with interfering ions and a visual color change technique from reddish to faint pink [636]. This study also demonstrates a morphology change by chemical surface leaching from nano-rod to nano-oval, proving the selectivity of Se nanomaterials to preferentially detect peroxide over other cellular cationic ions based on surface plasmon resonance. Moreover, spherical monoclinic SeNPs, synthesized using *B. subtilis*, exhibit high electrocatalytic activity in detecting H_2_O_2_ with a detection limit of 8 × 10^−8^ M, and show good adhesive ability and biocompatibility towards its substrates such as heme-containing proteins/enzymes [637]. Furthermore, colloidally stable SeNPs, produced using *B. pumilus* cell-free extract, enable the design of a low cost, sensitive, and reproducible H_2_O_2_ biosensor [638]. In addition, biogenic SeNPs, fabricated using the bacterial isolate *P. aeruginosa*, are used to design a biosensor for the visual assessment of the relative toxicity of a variety of chemicals, involving the inhibition of the bioreduction process of SeO_3_^2−^ in NP-treated bacterial culture supernatant, as a toxicity end-point [639]. This novel Se-bioassay could be easily applied to prescreen a plethora of environmental toxicants including nanostructures prior to intensive toxicity investigations. This adds up to the improved oxidase-like activity of CTS-SeNPs that provides a low-cost colorimetric method for Hg^2+^ detection with a detection limit of 0.12 μM, broadening, thus, the application of biogenic SeNPs in chemical sensor systems [640]. Lastly, Zhao et al. performed a green and controlled synthesis of SeNPs through a self-assembled method on molecular imprinting sites of zeolite-chitosan-TiO_2_ microspheres by coupling chitosan biosorption and TiO_2_ photocatalysis [641]. These NPs were successfully employed for dot-blot immunoassays with multiple native antigens for the prompt serodiagnosis of human lung cancer.

#### 7.8.2. Cellular Imaging

SeNPs have become one of the most prospective and potential tools for cancer diagnosis and therapy. For example, Korany et al. fabricated SeNPs capped with glutathione as a novel radio-platform for tumor imaging by studying the radiochemical yield of radioactive technetium-99m (^99m^Tc) in intravenous and intratumoral routes [346]. Sun et al. showed that luminescent Ru(II)-thiol SeNPs possess high tumor-targeted fluorescence imaging in HepG2 and HUVEC malignant cells while displaying improved antitumor efficacy and decreased systemic toxicity [642]. These functional SeNPs exhibit a well-defined, time-dependent increase in fluorescence intensity from 115 ± 17 a.u. after 0.5 h to 1171 ± 127 a.u. after 4 h. Huang et al. used SeNPs to build a smart drug-delivery nanoplatform to achieve simultaneous diagnosis, real-time monitoring, and therapy of cancer [643]. In this platform, epidermal growth factor receptor (EGFR) is used as the targeting molecule, gadolinium chelate as the magnetic resonance imaging contrast agent, polyamidoamine (PAMAM) and 3,3′-dithiobis (sulfosuccinimidyl propionate) as the response agents of intratumoral glutathione, 5-fluorouracil (^5^Fu) and cetuximab as drug payloads, and the pH as the release stimulus for the combined diagnosis and treatment of nasopharyngeal carcinoma (NPC) using SeNPs.

Vitamin C is a notable antioxidant human vitamin widely employed as a coating in NPs to prevent aggregation and achieve enhanced size and shape control and stabilization. Vitamin C-stabilized SeNPs (Vit C-SeNPs), labeled with ^99m^Tc for further in vivo studies on normal and solid tumors induced in mice, exhibit an enhanced antioxidant activity leading to improved uptake and retention by tumor cells [34]. Moreover, functional SeNPs are used to develop a siRNA-delivery system for vascular endothelial growth factor (VEGF), a known signaling molecule involved in cancer [644]. This design comprises two nanostructures, SeNPs@siRNA and G2/PAH-Cit/SeNPs@siRNA; the latter is a pH-sensitive delivery system able to improve siRNA loading. Importantly, the utilization of pH-sensitive functional SeNPs results in no lesions in major target organs, thereby offering a novel, safe, and promising cancer treatment. Furthermore, photodynamic SeNPs, modified with photosensitive and macrophage-targeting bilayers, control activated macrophages and quench the intracellular H_2_O_2_ and NO that are associated with chronic inflammation diseases [560]. The first layer of the photosensitive system consists of, principally, a conjugate of a photosensitizer (rose bengal, RB) and a thiolated chitosan (chitosan-glutathione), while the second layer is made by conjugating hyaluronic acid with folic acid using an ethylenediamine linker. In addition, the intense and long-lasting intrinsic fluorescence of individual SeNPs in the visible to near infrared range enables their application for real-time tracking and imaging in cells, without the need of any chemical tags or dyes [645]. Further, biogenic SeNPs, produced using a Se-tolerant strain of *S. maltophilia*, exhibit a higher ability to emit light (photoluminescence) than organic fluorophores, thus constituting potential markers for bioimaging and fluorescence lifetime imaging microscopy (FLIM) [219].

## 8. Translational Nanomedicine: Recent Progress, Emerging Challenges, and Future Prospects for Biogenic Selenium Nanoparticles

Nanotechnology has triggered tremendous developments that benefit different fields of science, especially biomedicine [646,647,648,649,650]. To improve human health, scientific discoveries, which start at “the bench” as a result of fundamental research, must be translated into practical applications by progressing to the clinical stage, named “bedside” [651]. The use of nanotechnology in molecular medicine offers a plethora of advantages, such as local and ultrafast strategies at the nanometer length scale (i.e., diffusion, intermixing, and sensor response), controlled and intensified physical and chemical processes, direct access to biomarkers, and real time studies [652]. Successfully translating nanomedicine agents from pre-clinical proof-of-concept to demonstrated therapeutic value in the clinic remains challenging since it is of paramount importance to develop more precise and better translatable nanodevices towards a patient-focused and disease-specific targeting from the outset [357,653]. Hence, nanosystems must grow substantially in safety and sophistication before focused smart nanomedicine can become a reality in which a single platform performs seamless processes ranging from ultrasensitive diagnosis to pinpoint therapy [654]. To enhance nanomedicine translation and performance, targeted therapies should employ a specific decision-making framework: correct tissue/exposure, correct target/efficacy, correct safety, correct patient, and correct commercial potential [655]. Moreover, translation strategies usually require innovation in the laboratory that must be supported by the pillars of evidence-based medicine for predictable regulatory outcomes.

Extensive research on engineered nanomaterials has led to the design of numerous nano-based formulations, i.e., nanomedicines, for theranostic applications with one of the significant benefits lying in the ability to formulate a drug without using dose-limiting toxic excipients present in many current marketed formulations, often enhancing tolerability and containing more active molecules to be administered to patients [655,656]. To date, SeNPs have demonstrated great preclinical applications in diagnosis and gene and drug delivery in cancer therapy owing, particularly, to their selective and effective accumulation in tumors through the enhanced permeability and retention (EPR) effect [119,657]. Nevertheless, there is a lack of research about the benefits of SeNPs in clinical settings, although they constitute a good candidate for advanced-stage clinical research due to their low toxicity and excellent biocompatibility [61,67].

The development of safer SeNPs with enhanced therapeutic efficacy in clinical settings requires a better understanding of the toxicity, possible side effects, and interaction with the biological environment. Before envisaging their application in humans, it is important to test their biosafety, degradation rate, long-term metabolic activity, pharmacokinetics and pharmacodynamics, and interaction with cells, organoids, etc. [658]. Nanomaterials usually interact with biomolecules in the physiological milieu, such as plasma proteins, which results in corona formation [656]. This corona can alter the NM stability, targeting ability, bio-identity, cellular uptake, dissolution properties, and change their biodistribution and in vivo toxicity [24,342,393,659,660]. Therefore, the nano–bio interface of SeNPs needs further assessments in terms of biomedical safety following rigorous methodologies, such as ADME-Tox as pointed out earlier.

The design and delivery of therapeutics to the brain has been an ongoing challenge in the treatment of brain tumors, especially due to the blood–brain barrier (BBB) that impedes reaching a proper local drug concentration [661,662,663,664]. Nanotechnology advances, such as the adequate surface functionalization, have improved the NP penetration across the BBB by receptor-mediated transcytosis [391,665]. For example, SeNPs coated with B6 peptide and functionalized with sialic acid (B6-SA-SeNPs) inhibit Aβ aggregation and pass the BBB, becoming a potential therapeutic nanovehicle to treat Alzheimer’s disease [666].

Although biogenic NPs are inexpensive, clean, non-toxic, and facile to produce via scalable processes, there has not yet been much work on their application in the fields of diagnostics and therapeutics, notably at the clinical stage [667,668,669,670,671]. The promising clinical applications of plant-synthesized metallic NPs have fueled a swelling interest switching from routine antioxidant and antimicrobial studies on trivial microbial lines to antibiotic-resistant pathogens and antitumor studies [672]. Furthermore, biogenic colloidal metallic NPs, especially those made of silver and gold, are found to be multifunctional theranostic agents [673]. However, more research should be directed towards developing facile and greener techniques for the large-scale fabrication of SeNPs using biological resources, as they hold potential applications in different aspects of nanomedicine, especially in combating cancer (Table 4). Indeed, selenium has been drawing great interest in the following clinical aspects: radioprotection of normal tissues, radiosensitizing in malignant tumors, antiedematous activity, prognostic impact, and effects on primary and secondary cancer prevention [674].

In a comprehensive review on strategies to enhance the success of clinical translation of cancer nanomedicines, van der Meel et al. listed four important factors: (i) stratification and selection of patients likely to respond to nanomedicine-based therapy, (ii) rational drug choice rather than opportunistic preferences, (iii) combination and multimodal therapies for synergistic effects, and (iv) empowering immunotherapy [653]. Hence, the engineering strategy of NPs, including those made of selenium, needs to reach important goals to boost the desirable effects and achieve transformation from formulation-driven research to disease-driven rational development. These goals include highly stable association of drug and carrier in systemic circulation, enhanced drug delivery to cancer cells, and controlled and prolonged release of active drugs in affected tissues [675]. Furthermore, the benefits of nanostructure-based diagnostics lie in their potentially higher sensitivity and selectivity compared to classical methods, thus enabling the earlier diagnosis of diseases resulting in enhanced resolution and sensitivity that will, ultimately, lead to novel, fast, convenient, and inexpensive screening, diagnostic, and therapeutic tools in medicine [652,676,677].

**Figure 12:**
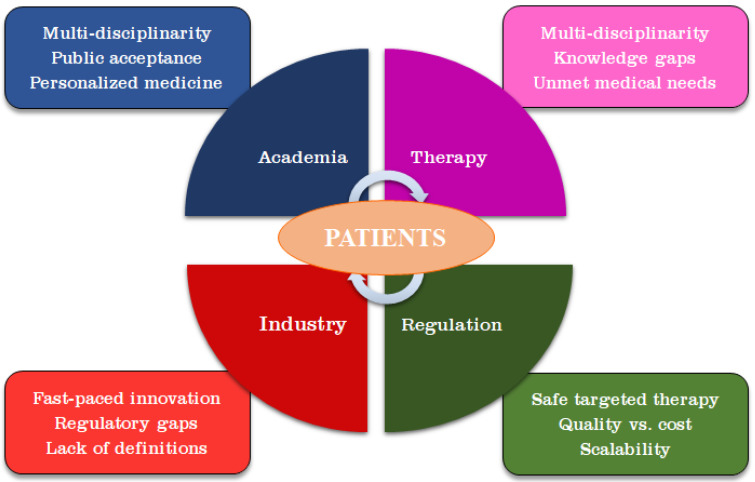
Stakeholders (academic researchers, clinicians and patients, industry, regulatory bodies) and the main challenges faced in the translation of nanomedicine.

Another challenge to overcome for the “next-generation nanomedicines” resides in the regulatory classification, because the lack of knowledge regarding biosafety and long-term effects of nanomaterials leads to a regulatory uncertainty and deficient standards and protocols for scale-up manufacturing and safe clinical uses [678,679]. This twilight zone in the nanomedicine market affects the effective collaborative work between stakeholders from industrial/academic R&D, professionals in the health system, regulatory bodies, and society (Figure 12). Moreover, classical methods of drug development and (non-)clinical assessments are expensive and encompass some uncertainties, delaying the advent of innovative approaches and the elucidation of safety concerns. For instance, the wrong information or the lack of full characterization of current nanoparticle-drug products leads to failures in follow-on versions, making further evaluation and manufacturing processes tedious before even envisaging any regulatory approval and marketing [679,680].

Advances in nanoscience and nanobiotechnology, combined with the call for personalized medicine based on nanotheranostics, have given rise to a cutting-edge, exciting, and fast-growing research area, where nanoscale Se formulations, including biogenic ones, are occupying a significant niche. It is necessary to precisely utilize engineered, nanotechnology-enabled solutions to face the challenges of the current drug delivery, imaging, therapeutics, and diagnostics procedures. Therefore, nanomedicine can lead to the next generation of biomedical breakthroughs by accelerating the translation of therapies with greater efficacy and reduced side effects into personalized/precision nanomedicine since innovative and clinically effective nanotherapeutics hold the potential to revolutionize nanoscale healthcare and pharmaceutical products and applications [681].

## 9. Conclusions and Perspectives

Selenium is a significant trace element which, in its elemental form or in the form of its various chemical species and Se-containing biomolecules, offers unique biological properties for the proper function of the body. Due to its high bioavailability, low toxicity, and affordability, nano-sized selenium has proven to be a proper nutritional supplement and an efficient theranostic agent. Based on these properties and the great versatility in their control thanks to green, biogenic routes for their synthesis and modification, SeNPs are emerging as a research hotspot in nanomedicine with the potential for promising benefits in clinical settings. Since biogenic SeNPs are free from toxic/hazardous components, they are well suited in biomedicine and therapeutics. Current findings highlight the outstanding physicochemical and biological properties of biogenic SeNPs that pave the way for extensive biomedical contributions, including pharmaceutical, therapeutic, imaging, and diagnostic applications. As such, SeNPs have been shown to combat cancer, pathogenic infections, diabetes, inflammatory syndromes, cardiovascular and neurological diseases, and drug-induced cyto- and geno-toxicity, among others. In addition to their antimicrobial and antiparasitic applications, SeNPs possess a potential utility in curbing viral outbreaks including the ongoing Covid-19 pandemic. Importantly, SeNPs constitute valuable nanoplatforms with multiple desired features for clinical translation. Thanks to the possibility of precise calibration and rational modification of their physicochemical properties, novel SeNPs are particularly attractive as therapeutic agents easily transportable in the organism and offering stability in the physiological microenvironment of target tissues. However, researchers are still in need of investigation into possible side effects due to the relatively narrow therapeutic window of Se compounds. Se nanoparticle-based diagnosis and therapy are in their early stages and preparing to progress into clinical trials. At this stage, many of these compounds could offer new mechanistic insights and pave the way towards the rational design of novel therapeutics following extensive analyses of structure–activity relationships (SARs). Thus, it is still necessary to accomplish further preclinical safety and selectivity studies before these new eco-friendly SeNPs are safely translated into clinical practice.

## Figures and Tables

**Table 1:** Classification of selenium compounds based on structural features. Adapted from Ref. [79] under Creative Commons Attribution license. 2012, MDPI AG.

Selenium Compound	Type
Selenoamino acids	Selenomethionine (SeMet)
	Methyl selenocysteine (MeSeCys)
	Selenocysteine (SeCys)
	Selenocystamine
Se-heterocyclic compounds	1,3-Selenazolin-4-one derivatives
	2-Phenyl-1,2-benzisoselenazol-3(2*H*)-one (ebselen)
	2,5-Bis(5-hydroxymethyl-2-selenienyl)-3-hydroxymethyl-*N*-methylpyrrole (D-501036)
	1,2-[Bis(1,2-benzisoselenazolone-3-(2*H*)-ketone)] ethane (BBSKE)
	2-Cyclohexylselenazolidine-4-(*R*)-carboxylic acid (ChSCA)
	2-Buthylselenazolidine-4-(*R*)-carboxylic acid (BSCA)
Selenocyanates	Isatin analogs
	Diphenylmethylselenocyanate
	1-4-Phenylenebis(methylene) selenocyanate (*p*-XSC)
	Temozolomide (TMZ)-Se
	5-Phenylcarbamoylpentyl selenocyanide (SelSA-2)
Selenides	Methylimidoselenocarbamates
	5-Phenylselenyl-methyl-2′-deoxyuridine (PhSe-T)
	5-Methylselenyl-methyl-2′-deoxyuridine (MeSe-T)
	β-Selenium amine derivatives
	Se,Se’-1,4-phenylenebis(1,2-ethanediyl) bisisoselenourea (PBISe)
Di-selenides	Bis(4-aminophenyl) diselenide
	Bis(5-phenylcarbamoylpentyl) diselenide (SelSA-1)
	Diselenodipropio nic acid (DSePA)
	2-Selenium-bridged β-cyclodextrin (2-SeCD)
Se(IV) compounds	Sodium selenite (Na_2_SeO_3_)
	Selenous acid (H_2_SeO_3_)
	Methyl selenic acid (MeSeA)
	Selenium dioxide (SeO_2_)

**Table 2:** Main types of selenoproteins and their functions.

Selenoproteins	Abbreviation	Localization	Function	Ref.
Glutathione peroxidase	GPX		Protection against oxidative stress. Catalytic reduction of H_2_O_2_	[75,246]
Cytosolic GPX1	GPX1	Cytoplasm, ubiquitous	Antioxidative defense	
Extracellular GPX	GPX3	Plasma and thyroid follicle	Anti-inflammatory activity	
Phospholipid GPX	GPX4	Mitochondrial membrane	Reduction of the phospholipid hydroperoxides. Membrane antioxidant	
Thioredoxin reductase	TrxR		Oxidoreductase activity with NADPH as the cofactor	[244]
Cytosolic TrxR	TrxR1	Mainly cytosolic, ubiquitous	Main antioxidant “weapon” at the cellular level. Inhibition of apoptosis and redox state of transcription factors	
Mitochondrial TrxR	TrxR2	Mitochondrial, ubiquitous	Regulation of cell proliferation	
Mitochondrial TrxR	TrxR3	Mainly mitochondrial, ubiquitous	Regulation of apoptosis and signaling pathway	
Iodothyronine deiodinase	DIO		Catalytic conversion of T4 and T3	[247]
Type I DIO	DIO1	Liver, lung, eyes, kidney, thyroid, pituitary, CNS *	Conversion of T4 into T3, T4 into rT3, and of T3 into T2	
Type II DIO	DIO2		Local (intracellular) production of T3 from T4 and T2 from rT3	
Type III DIO	DIO3	Placenta, fetus, liver, gravid uterus, fetal and neonatal brain, skin	Production of T2 from T4 and rT3 from T4	
Selenoprotein P	SePP	Thyroid and blood	Selenium transport and storage, endothelial antioxidant	[248]

* NADPH: nicotinamide adenine dinucleotide phosphate (reduced form of the redox coenzyme nicotinamide adenine dinucleotide phosphate); CNS: Central nervous system; T2: diiodothyronine; T3: triiodothyronine; rT3: reverse T3; T4: thyroxine.

**Table 3:** Antioxidant activity of biogenic SeNPs: Biosynthetic route, NP features (size and shape), methods used and main outcomes.

Biological System Used for Synthesis	Shape and Size of SeNPs	Antioxidant Measurement Technique	Antioxidant Activity	IC_50_ (μg/mL)	EC_50_ (μg/mL)	Ref.
*Bacillus* sp. MSh-1.	Spherical; 80–220 nm	DPPH and reducing power assays	RSA of 23.1 ± 3.4% Dose-dependent reducing power within a 0–200 μg·mL^−1^ concentration range	41.5 ± 0.9	N/A	[260]
*Saccharomyces cerevisiae*	Spherical; 50 nm	DPPH	RSA increase of 21.7–48.5% in a dose-dependent manner	N/A	N/A	[150]
*Bacillus* sp. EKT1	Spherical; 31–335 nm	DPPH	RSA of up to 56.5 ± 5.0% at 400 μg·mL^−1^	322.8	N/A	[493]
Quercetin and gallic acid	Bimetallic Ag-Se NPs capped by flavonoids and phenolics; 30–35 nm	ABTS, DPPH and MTT assays	59–62% T-AOC	30–66	N/A	[495]
Aqueous chitosan microspheres	Spherical; 36–95 nm	H_2_O_2_ levels; measurement of GSH, TBARS (MDA equivalent), GSH-Px, SOD and CAT	Increase in both intracorporeal Se retention and levels of GSH-Px, SOD and CAT; reduced levels of TBARS	N/A	N/A	[129]
*Emblica officinalis* extract	Spherical; 15–40 nm	DPPH and ABTS radical scavenging assays	Dose-dependent RSA, linear relationship with NP concentration	127.28 ± 3.73	DPPH: 15.67 ± 1.41 mg·mL^−1^ ABTS: 18.84 ± 1.02 mg·mL^−1^	[155]
*Lactobacillus casei* ATCC 393	50–80 nm	Cellular methods: T-AOC MDA T-SOD GSH-Px levels TrxR	Increased T-AOC, T-SOD, TrxR and GSH-Px Reduced MDA levels in serum and jejunum	N/A	N/A	[496]
*Pantoea* *agglomerans*	Spherical; 30–300 nm	Production of ROS using HUVEC fluorescence determined using a microplate reader at 485-nm excitation and 583-nm emission	Decrease in fluorescence resulting from the oxidation of the intracellular probe dichlorofluorescein (DCF)	N/A	N/A	[262]
*L. casei* ATCC 393	Spherical; capped with proteins and polysaccharides; 50–80 nm	H_2_O_2_ levels	Increased GPX activity, reduced MDA	N/A	N/A	[497]
		H_2_O_2_-induced oxidative damage model of human colon mucosal epithelial cells	Alleviated increase in ROS, reduced ATP and MMP Improved levels of Nrf2, HO-1, and NQO-1 proteins	N/A	N/A	[498]
*L. lactis* NZ9000	Spherical; 38–152 nm	H_2_O_2_ levels measurement; MDA T-SOD GPX	Alleviated IPEC-J2 cell oxidative damage caused by H_2_O_2_ Inhibition of intracellular ROS production	N/A	N/A	[499]
*Cordyceps sinensis* EPS conjugation	Amorphous and monoclinic; 80–125 nm.	ABTS and superoxide anion radical (O_2_${}^{•-}$) scavenging assays	Smaller SeNPs present high O_2_${}^{•-}$ scavenging ability. Se/P ratios (1:3, 1:1 and 4:3) had a higher ABTS^•+^ scavenging ability, and could reach 88.89%, 85.53% and 69.88%, respectively, at 0.2 mg·mL^−1^.	N/A	N/A	[148]
*Ephedra aphylla* extract	Spherical and tetragonal; 13.95–26.26 nm	DPPH assay	Lower activity of SeNPs a than the plant extract	0.213 and 0.296 mg·mL^−1^	N/A	[491]
Green tea extract and *Lycium barbarum* polysaccharides	Spherical and triangular; 83–160 nm	DPPH and ABTS assays	Strong, concentration-dependent DPPH-scavenging activity at 5–25 µM High antioxidant activity with low EC_50_ Dose-dependent inhibition of ABTS free radicals	N/A	22 µM	[500]
*Theobroma cacao* L. bean shell extract	Spherical; 1–3 nm	ABTS and FRAP assays	ABTS: 28.6 ± 0.1 mg TE/g FRAP: 12.4 ± 0.2 mg TE/g	N/A	N/A	[487]
Chitosan	Spherical; 102–104 nm	DPPH, ABTS and superoxide anion radical (O_2_${}^{•-}$) scavenging assays	DPPH: 83.06% at 0.5 mM ABTS: 74.33, 80.23 and 81.99% at 2 mM Superoxide: 25.20, 27.54, 31.44% at 1 mM	DPPH: 0.296, 0.306, 0.325, 0.370 mM ABTS: 1.314, 1.249, 1.143 and 1.101 mM	N/A	[489]
*Streptomyces minutiscleroticus*	Spherical; 100–250 nm	DPPH; Reducing power assay; T-AOC: Phosphomolybdenum method	All measurements increase in a dose-dependent manner T-AOC was more or less equal to the standard ascorbic acid	N/A	N/A	[316]
*Withania somnifera*	Spherical; 45–90 nm	DPPH	RSA increase in a dose-dependent manner in the range of 20–100 mg·mL^−1^ *	14.81 μg·mg^−1^	N/A	[160]
*Diospyros montana* leaf extract	Spherical; 4–16 nm	DPPH and FRAP assays	DPPH: color change from purple to pale yellow. RSA of 61.12% at 200 μg·mL^−1^ FRAP: color change from yellow to shades of green and blue	0.225	0.435	[490]
*Corbicula fluminea*	Spherical; 40–70 nm	DPPH, TEAC and FRAP of plasma assays.	DPPH RSA: 70, 77, 83, 79, and 53% at 1.5 mg·mL^−1^ *. Increase in a dose-dependent manner TEAC: highest RSA at 226 μmol Trolox/g sample FRAP: highest RSA at 150 μmol Fe^2+^/g sample	1.5 mg·mL^−1^ *	N/A	[488]
*Murraya koenigii*	Spherical; 50–150 nm	DPPH and Superoxide anion (O_2_${}^{•-}$) scavenging assay	Concentration-dependent RSA increase	25 and 50	N/A	[109]
Ginger plant *(Zingiber officinale)* extract	Spherical; 100–150 nm	DPPH	Dose-dependent RSA increase (disappearance of the purple color) SeNPs are free radical inhibitors or scavengers acting possibly as primary antioxidants	125	N/A	[501]

* We think these concentrations are in µg·mL^−1^. Abbreviations: ABTS: (2,2′-azino-bis(3-ethylbenzothiazoline-6-sulfonic acid); APX: Ascorbate peroxidase; CAT: Catalase; DAO: Diamine oxidase assay; DCF: Dichlorofluorescein; DPPH: 2,2-diphenyl-1-picrylhydrazyl; ABTS: 2,20-azino-bis (3-ethylbenzothiazoline-6-sulphonic acid); EC_50_: Effective concentration required to inhibit 50% of free radicals; EPS: Exopolysaccharides; FRAP: Ferric reducing antioxidant power; GSH: Glutathione; GSH-Px: Glutathione peroxidase; HUVEC: Human umbilical vein endothelial cells; IC_50_: Half maximal inhibitory concentration; IPEC-J2: Intestinal porcine enterocytes; MDA: Malondialdehyde assay; MMP: Mitochondrial membrane potential; MTT: 3-(4,5-dimethylthiazol-2-yl)-2,5-diphenyltetrazolium bromide; POX: Peroxidase; ROS: Reactive oxygen species; RSA: Radical scavenging activity; SOD: Superoxide dismutase; T-AOC: Total antioxidant capacity; TBARS: Thiobarbituric acid reactive substances; TEA: Trolox equivalent antioxidant; TEAC: Trolox equivalent antioxidant capacity; TrxR: Thioredoxin reductase; T-SOD: Total superoxide dismutase.

**Table 4:** Anticancer activity of several biogenic SeNPs against different cancer cell lines.

Biological System/Green Method	Shape and Size (nm)	Concentration/Dosage	Assay/Pathway	Cell Line *	Key Outcomes	Ref.
*Streptomyces bikiniensis*	Nanorods; 17 nm	10, 25, 50, and 100 μg·mL^−1^	MTT dye reduction assay	Hep-G2 (M) MCF-7 (M)	ID_50%_: 75.96 and 61.86 μg·mL^−1^, respectively. Loss of cell-to-cell contact, cell shrinkage, and formation of apoptotic bodies. Higher reduction of cell viability in Hep-G2 (42.3–86.9%) than in MCF-7 (37.5–69.1%).	[521]
*Cassia auriculata* leaf extract	10–20 nm	0.5 to 150 μg·mL ^−1^	MTT assay	HL-60 (M) Vero cell line (NM)	Antileukemia activity in a dose-dependent manner with a CC_50_: 7.01 μg·mL^−1^ (HL-60) and 109.13 μg·mL^−1^ (Vero cells)	[162]
Garlic (*Allium sativum*) clove extract	Spherical; 40–100 nm	15, 30, 60 and 90 μg·mL^−1^	MTT assay	Vero cell line (NM)	CC_50_ of 31.8 ± 0.6 μg·mL^−1^	[522]
Chitosan (CTS-SeNPs) and *Pleurotus ostreatus* fermented fenugreek (SeNPs-AEFFP)	Spherical; CTS-SeNPs: 45 nm SeNPs-AEFFP: 11.8 nm	CTS-SeNPs: 1.187–38 μg·mL^−1^ SeNPs-AEFFP: 0.594–19 μg·mL^−1^ NPs exposed to γ-ray doses of 60 and 15 kGy, respectively, against EaC and CaCo-2.	Trypan blue (0.5%) assay	EaC (M) CaCo-2 (M)	For EaC: CTS-SeNPs: IC_50_ = 23.12% SeNPs-AEFFP: IC_50_ = 7.21% For CaCo-2: CTS-SeNPs: IC_50_ = 25.32% SeNPs-AEFFP: IC_50_ = 8.57% SeNPs exhibit a concentration-dependent repression against EaC and CaCo-2 and a selective cytotoxic effect.	[112]
*E. coli*	Spherical, elliptical and nanorods; 60 nm	20, 60, and 100 μg·mL^−1^	MTT and addition of DMSO. Caspase 3-involved apoptosis pathway.	A549 (M) IMR-90 (NM)	Cell viability of ∼70%, ∼45% and ∼25%, high ROS generation and elevated caspase-3 activity.	[523]
*B. licheniformis* JS2	Spherical; 110 nm	1, 2, 4, 6, 50, or 200 μg Se/mL for 24 h	Colorimetric XTT assay, activation of caspases 3 and 7, DMSO treatment and hemolysis assays	LNCaP-FGC (M)	Overexpression of TNF and IRF1, reducing the expression of androgen receptors. SeNPs decrease the cell viability regardless of apoptosis and necrosis. SeNPs induce cell death through neither apoptosis nor necrosis.	[524]
*S. minutiscleroticus* M10A62	Spherical; 10–250 nm	50–100 μg·mL^−1^	MTT	Hep-G2 (M) HeLa (NM)	50 μg·mL^−1^ concentration of SeNPs was required for 99.5% HepG2 growth inhibition and 100 μg·mL^−1^ for 100% growth inhibition of HeLa.	[316]
*B. licheniformis* JS2	110 nm	Minimum of 2 μg Se/mL	Real-time qPCR analysis; confocal microscopy; treatment with cytochalasin D	PC-3 (M)	ROS mediated necroptosis of PC-3 cells independent of RIP3 and MLKL and regulated by a RIP1 kinase. Increased expression of necroptosis associated to TNF and IRF1	[295]
Fenugreek seed extract	Amorphous; 50–150 nm	25, 50, 75, and 100 μg·mL^−1^ for 24 h	MTT assay	MCF-7 (M)	SeNPs augment the cytotoxicity of doxorubicin and induce MCF 7 cell death through apoptosis.	[525]
*Idiomarina* sp. PR58-8	Spherical; 150–350 nm	5–100 μg·mL^−1^ for 24 h	MTT assay, ROS assay, apoptotic index assay, Western blot assay.	HaCaT (NM) HeLa cells (M)	Caspase-dependent apoptosis in HeLa cell lines: decrease in expression of pro-caspase 3. SeNPs exhibited dose-dependent cytotoxicity with only 3% viability at 100 μg·mL^−1^.	[526]
*Acinetobacter* sp. SW30	Nanospheres and crystalline nanorods of 78 nm. Polygonal-shaped SeNPs of 79 nm.	0–100 μg·mL^−1^	MTT assay	4T1 (M) MCF-7 (M) NIH/3T3 (NM) HEK293 (NM)	Antiproliferative activity.	[527]
*Bacillus* sp. MSh-1	Spherical; 80–220 nm	10, 20, 50 and 100 μg·mL^−1^	MTT assay and gelatin zymography	HT-1080 (NM)	SeNP dose of 100 μg·mL^−1^ decreases the viability of the cell line to 50%, whereas a lower dose (10 μg·mL^−1^) shows a low level of cytotoxicity with a viability of more than 80%.	[528]
*Asteriscus graveolens* extract	Spherical; 20 nm	25–125 mg·mL^–1 ‡^ for 24 h	MTT assay, flow cytometry analysis, measurement of ROS (conversion of DCFH-DA to DCFH); measurement of MMP and lipid peroxidation	HepG2 (M)	Cell viability (IC_50_): 51.8% at 3.98 μg·mL^−1^. SeNPs inhibit the growth of HepG2 cells mainly by induction of apoptosis. They also significantly and rapidly increase the ROS level.	[285]
*Moringa oleifera* extract	Spherical; 23–35 nm Polygonal; 25–45 nm	N/A	MTT assay	CaCo-2 (M) HepG2 (M) MCF-7 (M)	IC_50_: 50.3% at 392.57 μg·mL^−1^	[156]
*Penicillium corylophilum*	Spherical; 29.1–48.9 nm	1000, 500, 250, 125, 62.5 and 31.25 ppm incubated in 5% CO_2_ at 37 °C for 24 h	MTT assay	WI-38 (NM) CaCo-2 (M)	IC_50_: 171.8 ppm (Wi 38) and 104.3 ppm (CaCo-2)	[502]
Hawthorn fruit extract	113 nm	0, 5, 10 and 20 μg·mL^−1^ for 24 h	MTT assay; flow cytometric analysis; ROS detection; MMP measurement; Western blot assay	HepG2 (M)	IC_50_: 19.22 ± 5.3 μg·mL^−1^	[529]
*L. casei* 393	Spherical; 50–80 nm	4, 8, and 16 μg·mL^−1^ for 12 h	RT-PCR; mRNA expression levels of Bax, caspase 3, p53 and bcl-2.	HepG2 (M) NCM460 (NM)	Endocytosis of SeNPs induces cell death by reducing the viability of HepG2, increasing mRNA levels of caspase 3, Bax, and p53, and reducing mRNA expression of bcl-2.	[497]
*Carica papaya* latex	Spherical; 70 nm	5, 10, 15, 20, 25, 30, 35, 40, 45, and 50 μg·mL^−1^ for 48 h	MTT assay	HBL100 (NM) MDA-MB-231 (M)	IC_50_ (HBL100): 50 μg·mL^−1^ IC_50_ (MDA-MB-231): 34 μg·mL^−1^	[530]
*Spermacoce hispida* aqueous leaf extract (Sh-SeNPs) + S-allyl glutathione conjugation (SAG-Sh-SeNPs)	Sh-SeNPs: aggregation SAG-Sh-SeNPs: spherical; 50 nm	HepG2: 1.88, 3.75, 7.50, 15.00 and 30.00 μg·mL^−1^ for 24 h Vero cells: 3.7–60.0 μg·mL^−1^ for 48 h	MTT assay; determination of intracellular ROS production and MMP; cell cycle analysis by flow cytometry; DNA fragmentation assay; apoptosis determination by acridine orange/ethidium bromide staining	HepG2 (M) Vero cells (NM)	IC_50_ (Sh-SeNPs): 30.0 μg·mL^−1^ IC_50_ (SAG-Sh-SeNPs): 18.7 μg·mL^−1^ SAG-Sh-SeNPs induce cell cycle arrest at sub-G1 phase and further lead to apoptosis. The NPs increase ROS levels, disrupt MMP, initiate DNA fragmentation and decrease the endogenous levels of antioxidants, such as GSH, superoxide dismutase, catalase and GSH peroxidase.	[531]
*Undaria pinnatifida* polysaccharides	Spherical; 44–92 nm (average of 59 nm)	N/A	MTT assay; flow cytometry analysis; Annexin-V-FLUOS staining assay; measurement of ROS levels; MMP evaluation	A375 (M) CNE2 (M) Hep G2 (M) MCF-7 (M)	IC_50_ values ranging from 3.0 to 14.1 μM. Apoptosis with the involvement of oxidative stress and mitochondrial dysfunction.	[532]
*Ceropegia bulbosa* tuber’s aqueous extracts	Spherical; 55.9 nm	0, 5, 10.0, 15.0, 20.0, 25.0, 30.0, 35.0, 40.0, 45.0, 50.0 μg·mL^−1^	MTT assay	MDA-MB-231 (M) HBL-100 (M)	IC_50_ (MDA-MB-231): 34 μg·mL^−1^ for 48 h. IC_50_ (HBL-100): more than 50 μg·mL^−1^ for 48 h.	[506]
*Diospyros montana* leaf extract	4–16 nm	50, 150, 250, 350 μg·mL^−1^	MTT assay	MCF-7 (M)	IC_50_: 80.83 μg·mL^−1^, SeNPs enhance the cytotoxicity.	[490]
Chitosan decoration	Spherical; 50 nm	10, 50, 100 μM	WST-1 assay	HepG2 (M)	SeNPs decrease the cell viability to 76.63, 63.31 and 56.34% and inhibit the growth of HepG2 cells in a time- and dose-dependent manner.	[489]
*Ephedra aphylla* extract	Spherical and tetragonal; 13.95–26.26 nm	N/A	MTT assay	HepG-2 (M) MCF-7 (M) HCT-116 (M) HeLa (M) PC3 (M) HEp2 (M)	IC_50_ (HePG-2): 7.56 ± 0.60 μg·mL^−1^ IC_50_ (MCF-7): 15.65 ± 1.40 μg·mL^−1^ IC_50_ (HCT-116): 10.02 ± 0.90 μg·mL^−1^ IC_50_ (HeLa): 9.23 ± 0.80 μg·mL^−1^ IC_50_ (PC3): 18.63 ± 1.50 μg·mL^−1^ IC_50_ (HEp2): 12.10 ± 1.20 μg·mL^−1^	[491]
Lentinan (LNT, denatured β-glucan)	Spherical; 28 nm	N/A	MTT assay	HeLa (M)	IC_50_ of three complexes Se/s-LNT-1, Se/s-LNT-2, Se/s-LNT-3 were estimated to be 85, 37, and 19 μM. SeNPs with small, uniform size greatly enhanced the antitumor activity and bioavailability.	[533]
Quercetin and gallic acid	Bimetallic Ag-Se NPs of 30–35 nm and capped by flavonoids and phenolics.	50, 100, 250 and 500 μg·mL^−1^	MTT assay	DL (M)	The viability of DL cells was 20% at 50 μg·mL^−1^ of Ag-SeNPs, while at 100 μg·mL^−1^, it was reduced to 15%. The Ag-SeNPs showed strong anticancer activity at a lower concentration.	[495]
Cationic pullulan (CP)	Spherical and microflowers; 50 nm	N/A	MTT assay; Annexin-V-FITC and propidium iodide (PI) staining	L929 (M) KB (M)	IC_50_: 0.060 μM. The early- and late-stage apoptotic rates of KB cells treated with doxorubicin only reached 0.52% and 4.64%, respectively, and the highest induction of 55.8% was arrested in the necrosis rate.	[534]
Walnut peptides	Spherical; 89.22 nm	200 μL·mL^−1^	MTT assay; POM, flow cytometry; MMP assay; nuclear morphology analysis by Hoechst 33258; measurement of ROS production; DNA fragmentation assay; caspase activity assay; Western blot assay	HL-7702 (L02) (M) MCF-7 (M) SGC-7901 (M) A549 (M) PC3 (M) HeLa (M)	MCF-7 cells were the most sensitive to SeNPs. The apoptosis-inducing activity was proved by the accumulation of S-phase cell arrest, nuclear condensation, and DNA breakage. The intrinsic signal pathway was through the activation of FADD and caspases 3, 8, and 9, in combination with the MMP depletion and ROS generation.	[535]

* Cell lines: 4T1: murine mammary carcinoma cell line; A375: human melanoma cell line; A549: human lung carcinoma cells; CaCo-2: human colorectal adenocarcinoma; CNE-2: human nasopharyngeal carcinoma cell line; DL: Dalton’s lymphoma (mice); EAC: Ehrlich ascites carcinoma (mice); HaCaT: normal human epidermal keratinocyte cell line; HBL100: human epithelial breast cell line (non-malignant); HCT-116: human colorectal carcinoma cell line; HDF: primary human dermal fibroblasts (non-malignant); HEK293: human embryonic kidney cell line (non-malignant); HeLa: human cervical carcinoma cell lines; HEp2: human epithelial carcinoma cell line; Hep-G2: human hepatic carcinoma cell line; HL-60: human leukemia cells; HL-7702 (L02): human liver cell line (non-malignant); HT-1080: human fibrosarcoma cell line; IMR-90: normal lung fibroblast cells (non-malignant); KB: human cervical carcinoma cell line; L292: fibroblast cell line (mice, non-malignant); LNCaP-FGC: human prostate epithelial carcinoma cell line; MCF-7: human breast adenocarcinoma cells; MDA-MB-231: human breast adenocarcinoma cell line; NCM460: human colon mucosal epithelial cell line (non-malignant); NIH/3T3: murine embryonic fibroblasts (non-malignant); PC3: human prostate adenocarcinoma cell line; PC-3: human prostate adenocarcinoma cell line; SGC-7901: human gastric cancer cell line, Vero cell lines (monkey, kidney, non-malignant); WI-38: human embryonic lung cell line (non-malignant); M: malignant; NM: non-malignant. Abbreviations: CC_50_: Cytotoxicity concentration; DCFH: 2′,7′-Dichlorodihydrofluorescein; DCFH-DA: 2′,7′–dichlorodihydrofluorescin diacetate; DMSO: Dimethylsulfoxide; DNA: Deoxyribonucleic acid; FADD: Fas associated via death domain; FITC: Fluorescein isothiocyanate; IC_50_: Half maximal inhibitory concentration; IRF: Interferon regulatory factor; MLKL: Mixed lineage kinase domain such as pseudokinase; MMP: Matrix metalloproteinases; mRNA: Messenger ribonucleic acid; MTT: 3-(4,5-dimethylthiazol-2-yl)-2,5-diphenyltetrazolium bromide; POM: Polarized optical microscopy; RIP: Receptor interacting serine/threonine kinase; ROS: Reactive oxygen species; RT-(q)PCR: Real-time (quantitative) polymerase chain reaction; TNF: Tumor necrosis factor; WST-1: 4-[3-(4-Iodophenyl)-2-(4-nitro-phenyl)-2*H*-5-tetrazolio]-1,3-benzene sulfonate; XTT: sodium 3′-[1-(phenylaminocarbonyl)-3,4-tetrazolium]-bis (4-methoxy6-nitro) benzene sulfonic acid hydrate. ^‡^ We think the concentration is in µg·mL^−1^.
